# Synthesis of electrophile-tethered preQ_1_ analogs for covalent attachment to preQ_1_ RNA

**DOI:** 10.3762/bjoc.21.35

**Published:** 2025-03-04

**Authors:** Laurin Flemmich, Ronald Micura

**Affiliations:** 1 Institute of Organic Chemistry, Center for Molecular Biosciences Innsbruck (CMBI), Innrain 80-82, 6020 Innsbruck, Austria

**Keywords:** deazapurines, heterocycles, pyrrolopyrimidines, queuosine, riboswitches, ribozymes, RNA alkylation, RNA labelling

## Abstract

The preQ_1_ cIass-I riboswitch aptamer can utilize 7-aminomethyl-7-deazaguanine (preQ_1_) ligands that are equipped with an electrophilic handle for the covalent attachment of the ligand to the RNA. The simplicity of the underlying design of irreversibly bound ligand–RNA complexes has provided a new impetus in the fields of covalent RNA labeling and RNA drugging. Here, we present short and robust synthetic routes for such reactive preQ_1_ and (2,6-diamino-7-aminomethyl-7-deazapurine) DPQ_1_ ligands. The readily accessible key intermediates of preQ_0_ and DPQ_0_ (both bearing a nitrile moiety instead of the aminomethyl group) were reduced to the corresponding 7-formyl-7-deazapurine counterparts. These readily undergo reductive amination to form the hydroxyalkyl handles, which were further converted to the haloalkyl or mesyloxyalkyl-modified target compounds. In addition, we report hydrogenation conditions for preQ_0_ and DPQ_0_ that allow for cleaner and faster access to preQ_1_ compared to existing routes and provide the novel compound DPQ_1_.

## Introduction

**Scheme 1:**
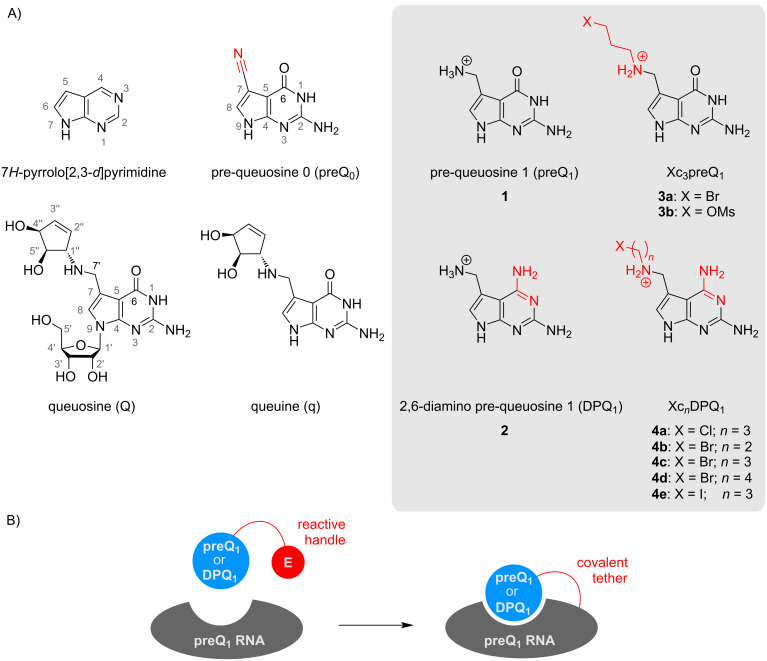
A) Chemical structures of hypermodified nucleobase queuine and nucleoside queuosine (Q) occurring as natural tRNA modifications. Purine ring numbering is indicated in grey. The synthetic targets of this study are highlighted in grey: Natural riboswitch ligand pre-queuosine **1** (preQ_1_), a novel preQ_1_ analog with altered base-pairing properties (2,6-diamino-pre-queuosine **2**, DPQ_1_) and haloalkyl-modified preQ_1_ and DPQ_1_. B) The ligand classes of Xc*_n_*preQ_1_ and Xc*_n_*DPQ_1_ allow specific formation of covalent small molecule–RNA complexes as has been recently demonstrated (see ref. [4]). Electrophile (E).

Pre-queuosine 1 (preQ_1_) is a biosynthetic precursor of the hypermodified nucleoside queuosine (Q) that is found in the wobble position of bacterial as well as eukaryotic aspartyl-, asparaginyl-, histidyl- and tyrosyl-tRNA isoacceptors bearing the G_34_U_35_N_36_ anticodon motif [1]. Like other tRNA anticodon modifications, queuosine has been shown to increase translational fidelity and efficiency [2]. Structurally, queuosine and preQ_1_ (compound **1**, Scheme 1A) belong to the 7-deazapurine family, which contain a pyrrolo[2,3-*d*]pyrimidine core. A rich pool of natural 7-deazapurine products has been identified, often (apparently) sharing a common biosynthetic pathway. Their functions are diverse; while some have been identified as having antifungal or antibiotic properties, others expand the chemical diversity and thus the functional sophistication of ribonucleic acids, as in the case of Q [3].

In most bacteria, Q biosynthesis is tightly regulated by riboswitches, which are highly structured RNA elements located mostly in the 5’-leader of messenger RNA. PreQ_1_ riboswitches sense the cellular concentration of preQ_1_ and regulate the expression of downstream located genes associated with the biosynthesis or transport of Q in a feedback-like manner. Binding of PreQ_1_ to the mRNA causes the riboswitch to commit an altered folding pathway, which affects the transcription or translation of the mRNA and results in altered transcript and/or protein levels [5].

In recent years, there has been a growing interest in the modification of preQ_1_. preQ_1_ derivatives, preQ_1_ analogs and mimics of preQ_1_ have greatly expanded our understanding of preQ_1_-binding biomolecules, such as riboswitches [6–8] or the queuosine biosynthetic enzyme machinery [9–11]. Recently, even the self-methylation activity of a preQ_1_ riboswitch has been discovered with a methylated preQ_1_ derivative acting as a ribozyme cofactor [12]. Moreover, these analogs have found utility in several biotechnological applications, including the identification of queuosinylation sites in cellular RNA [13], RNA and DNA labeling [14–15], and mRNA photocaging [16]. The latter applications rely on the promiscuity of the tRNA-modifying enzyme *tRNA-guanine transglycosylase* (TGT), which can incorporate functionalized preQ_1_ congeners into oligonucleotide strands at specific recognition sites.

In addition, the potential of modified preQ_1_ for protein enzyme-independent RNA labeling has also been demonstrated [12,17]. In a recent study [4], sequence-specific RNA–small molecule crosslinking (Scheme 1B) was achieved in vitro and in living cells using rationally designed electrophile-tethered derivatives of preQ_1_ (**1**) and its Watson–Crick diamino-faced counterpart DPQ_1_ (**2**, Scheme 1A). These ligands (compound classes **3** and **4**, Scheme 1A) were tailored to target a conserved guanine nucleobase within a natural preQ_1_-binding mRNA domain, namely the preQ_1_ class-I riboswitch (preQ_1_-I) from *Thermoanaerobacter tengcongensis*. By rigorously analyzing the high-resolution structures available for this ligand–RNA complex, the approach exploits the natural, sequence-inherent reactivity hotspots of RNA and thus avoids the use of highly electrophilic warheads otherwise typically employed in RNA-small molecule crosslinking [18–21]. Instead, primary alkyl halides (or mesylates, Scheme 1, in particular compounds **3a**, **3b** and **4c**) were found to be potent yet mild alkylators that minimize off-target reactivity, while providing reasonably fast labeling kinetics and up to quantitative conversion under quasi-physiological conditions [4].

Obviously, the rapid dissemination and widespread acceptance of such labeling methods depend on fast and simple access to the small molecule probes. Here, we report efficient synthetic routes to haloalkyl- and mesylate-modified preQ_1_ **3a** and **3b**, the corresponding variants with different Watson–Crick face of DPQ_1_ (**4a–e**), as well as to the nucleobase precursors preQ_1_ (**1**) and DPQ_1_ (**2**), respectively.

## Results and Discussion

### Synthesis of preQ_1_ and DPQ_1_

**Scheme 2:**
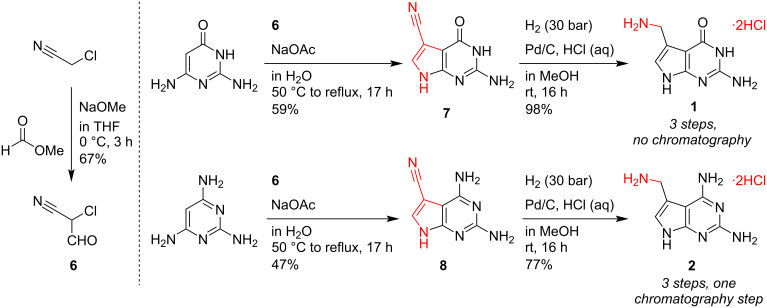
Three-step syntheses of preQ_1_ (**1**) and DPQ_1_ (**2**). For the synthesis of m^6^preQ_1_ (**16**) see Supporting Information File 1.

Several synthetic strategies towards preQ_1_ and its derivatives have been reported [22–26]. Among these reports, various silylation and protection strategies have been employed to address the poor solubility of preQ_1_ (and analogs) in organic solvents [9,22–23,25–29]. Herein, we report an optimized three-step protocol, free of protecting groups and time-consuming purification steps, that provides preQ_1_ in 43% overall yield with a purity of >98% (Scheme 2). The approach is based on the cyclocondensation reaction between 2-chloro-3-cyanopropan-1-al (**6**) (itself obtained from chloroacetonitrile and methyl formate) and 2,6-diaminopyrimidin-4(*3H*)-one to afford preQ_0_ (**7**), as originally reported by Townsend et al. [30]. The next step, namely the reduction of the nitrile moiety by hydrogenation is critical and notoriously difficult due to the low reactivity of this group in preQ_0_ [26]. We solved this problem by applying strongly acidic protic conditions [31] together with a 7-fold increase in hydrogenation pressure (30 bar); this resulted in an almost quantitative conversion and pure preQ_1_ (**1**) in the form of its dihydrochloride salt which was isolated after a simple filtration step.

Using the same approach, we were able to prepare the novel 2,6-diamino preQ_1_ analog **2** (DPQ_1_) by hydrogenation of **8** (DPQ_0_ [32]) (Scheme 2). In this case, however, a final purification step (by reversed-phase chromatography) was required. Notably, using the hydrogenation conditions described here, we were also able to streamline our previously reported 7-step synthesis of *O*^6^-methyl preQ_1_ (**16**, m^6^preQ_1_) [28]. The direct reduction of the precursor *O*^6^-methyl preQ_0_ (**15**, m^6^preQ_0_) was possible, eliminating the need for the previously introduced protection/solubility concept, which shortened the synthetic route to only four steps (Supporting Information File 1).

### Synthesis of preQ_1_ and DPQ_1_ derivatives with electrophilic handles

**Scheme 3:**
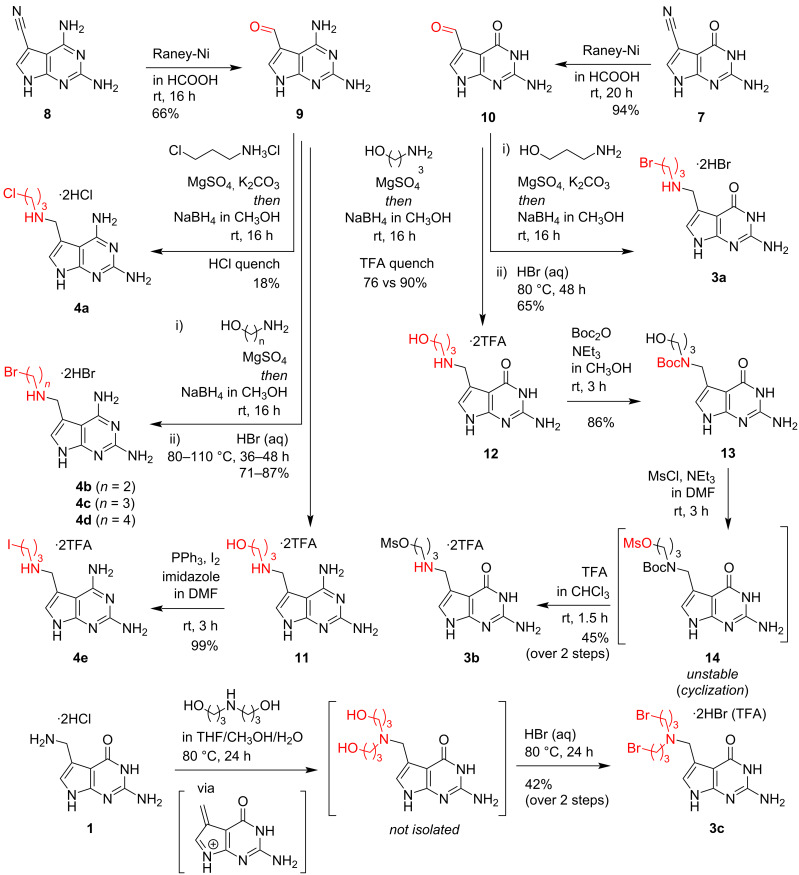
Syntheses of haloalkyl- and mesyloxyalkyl-modified preQ_1_ as and DPQ_1_ ligands.

For the synthesis of haloalkyl- and mesyloxyalkyl-modified preQ_1_ and DPQ_1_ ligands **3a**,**b** and **4a–e** (for target structures see Scheme 1), a divergent synthetic route was sought that provided flexibility with respect to linker length and nature of the electrophile. We thus identified aldehydes **9** and **10** as suitable branching points, which were easily derivatized to their aminomethyl-modified preQ_1_ analogs by reductive amination (Scheme 3). Their syntheses by Raney-Ni reduction of nitriles **7** and **8**, previously described by Gangjee and co-workers [33], proceeded cleanly in our hands.

In the case of compound **4a**, the chloropropyl moiety was directly installed by reductive amination of **9** with 3-chloropropylamine hydrochloride under basic conditions. A two-step reaction sequence, however, was necessary to generate derivatives **4b–e** and **3a**. First, the alkyl handles bearing a primary hydroxy group were introduced and then converted into the electrophile of choice. More specifically, to furnish compounds **4b–d**, precursor **9** was treated with the corresponding amino alcohols in the presence of a desiccant. The imines formed were subjected to mild reduction with methanolic sodium borohydride. Upon purification by reversed-phase chromatography using aqueous hydrobromic acid as eluent (0.5% in H_2_O), a considerable fraction of the alcohols was already converted to the desired bromides **4c**,**d**. Only in the case of **4b**, no deoxygenative bromination was observed, and the alcohol intermediate was isolated in pure form. In both cases, quantitative bromination was achieved by heating the compounds in concentrated aqueous hydrobromic acid, which after evaporation afforded the pure compounds **4b–d**.

To generate iodide **4e**, alcohol **11** was isolated and subjected to Appel conditions in DMF, using elemental iodine as the halogen source. Notably, we were not able to efficiently generate the corresponding bromides with the same strategy.

The preQ_1_ derivative **3a** was synthesized in a 2-step reaction sequence analogous to the DPQ_1_ derivative **4b**, while four steps were required to obtain the corresponding mesylate **3b**. Similar to **11**, compound **12** was isolated as its trifluoroacetate salt. Selective Boc protection of the aliphatic amine gave **13**, which was selectively *O*-mesylated to give compound **14**. Compound **14** was found to slowly undergo intramolecular cyclization by displacement of the mesyl group to give a six-membered cyclic carbamate, a reactivity that has been described earlier [34]. Thus, care was taken to quickly isolate compound **14** and use it immediately in the next step. Deblocking of the secondary amine by treatment with trifluoroacetic acid afforded **3b** in almost quantitative yield.

The bis(3-bromopropyl)-modified ligand **3c** was generated by heating preQ_1_ together with bis(3-hydroxypropyl)amine. It is noteworthy that the amine exchange reaction is thought to proceed via a purine methide intermediate [11]. Subsequent treatment of the diol with aqueous hydrobromic acid provided **3c**.

## Conclusion

We have developed a divergent synthesis of 7-aminomethyl-7-deazapurines (preQ_1_ and DPQ_1_) with various electrophilic handles extending the aminomethyl moiety. These derivatives are capable of covalent tethering to the preQ_1_-I RNA aptamer. This aptamer occurs naturally in mRNA riboswitches in bacteria and is involved in ligand-dependent gene regulation. Therefore, this riboswitch (like others) has become an attractive target for drug design.

To date, most known RNA–small molecule binders interact in a non-covalent manner. The compounds presented here are part of our research program to tailor non-covalent RNA–small molecule ligands to their covalent counterparts. While “covalent drugs” have become a leading principle in medicinal chemistry in the “protein world” [35–36] – approximately 30% of all FDA-approved drugs form a covalent bond with their target protein – this concept is underexplored in the field of RNA drugging [37]. Recent studies suggest that the validation of RNA–small molecule interactions [38–40], drug efficacy or the identification of off-target effects of approved drugs on the transcriptome [41–42] could greatly benefit from covalency. We believe that these exciting new research directions will be furthered by the efficient synthetic routes to covalent RNA binders presented here.

## Experimental

**General procedure for reductive aminations (compounds 3a, 4b–d, 11, and 12).** Aldehyde **9** or **10** (50.0 mg, 282 µmol) was suspended in methanol (1.3 mL). Anhydrous magnesium sulfate (340 mg, 2.82 mmol, 10 equiv) and the respective amino alcohol (2.82 mmol, 10 equiv) were added. The mixture was sonicated for 30 minutes and subsequently stirred at room temperature for 16 h. After cooling to 0 °C, sodium borohydride (96.1 mg, 2.54 mmol, 9 equiv) was added in portions over the course of 1 h. The mixture was stirred for additional 2.5 h at room temperature. Afterwards, the volatiles were removed under reduced pressure and the residue was taken up in dilute aqueous acid (for composition see individual experiments in Supporting Information File 1; compounds **3a**, **4b–d**, **11**, and **12**). If insolubles were present after pH 1–2 was reached, the suspension was filtered. Purification is described in Supporting Information File 1 for the individual compounds **3a**, **4b–d**, **11**, and **12**.

## Supporting Information

File 1 Experimental part, HPLC analysis of preQ1 and NMR spectra.

## Data Availability

All data that supports the findings of this study is available in the published article and/or the supporting information of this article.
