# Supplementary material for: Synthesis of electrophile-tethered preQ1 analogs for covalent attachment to preQ1 RNA
Source: Beilstein J Org Chem. 2025 Mar 4;21:483–9. doi: 10.3762/bjoc.21.35 (PMC11897656; doi:10.3762/bjoc.21.35)
Supplement: File 1 — Experimental part, HPLC analysis of preQ1 and NMR spectra. [file Beilstein_J_Org_Chem-21-483-s001.pdf]

BEILSTEIN JOURNAL OF ORGANIC CHEMISTRY

Supporting Information

for

Synthesis of electrophile-tethered $preQ_1$ analogs for covalent attachment to $preQ_1$ RNA

Laurin Flemmich and Ronald Micura

Beilstein J. Org. Chem. **2025,** *21,* 483–489. doi:10.3762/bjoc.21.35

Experimental part, HPLC analysis of preQ1 and NMR spectra

License and Terms: This is a supporting information file under the terms of the Creative Commons Attribution License (https://creativecommons.org/licenses/by/4.0). Please note that the reuse, redistribution and reproduction in particular requires that the author(s) and source are credited and that individual graphics may be subject to special legal provisions.
The license is subject to the *Beilstein Journal of Organic Chemistry* terms and conditions: (https://www.beilstein-journals.org/bjoc/terms)

Table of Contents

1. Synthetic procedures **S2**

General S2
2-Chloro-3-oxopropanenitrile (**6**) S2
preQ$_0$ (**7**) S2
preQ$_1$ dihydrochloride (**1**) S2
DPQ$_0$ (**8**) S3
DPQ$_1$ dihydrochloride (**2**) S3
7-Formyl-7-deaza-2,6-diaminopurine (**9**) S3
7-Formyl-7-deazaguanine (**10**) S3
7-(*N*-(3'-Chloropropyl)aminomethyl)-7-deaza-2,6-diaminopurine (**4a**) S4
7-(*N*-((2'-Bromoethyl)aminomethyl)-7-deaza-2,6-diaminopurine dihydrobromide (**4b**) S4
7-(*N*-(3'-Bromopropyl)aminomethyl)-7-deaza-2,6-diaminopurine (**4c**) S4
7-(*N*-(4'-Bromobutyl)aminomethyl)-7-deaza-2,6-diaminopurine (**4d**) S5
7-(*N*-(3'-Hydroxypropyl)aminomethyl)-7-deaza-2,6-diaminopurine trifluoroacetate salt (**11**) S5
7-(*N*-(3'-Iodopropyl)aminomethyl)-7-deaza-2,6-diaminopurine trifluoroacetate salt (**4e**) S5
7-(*N*-(3'-Bromopropyl)aminomethyl)-7-deazaguanine dihydrobromide (**3a**) S6
7-(*N*-(3'-Hydroxypropyl)aminomethyl)-7-deazaguanine trifluoroacetate salt (**12**) S6
7-(*N*-(3'-Hydroxypropyl)-*N*-(tert-butyloxycarbonyl)aminomethyl)-7-deazaguanine (**13**) S6
7-(*N*-(3'-Mesyloxypropyl)aminomethyl)-7-deazaguanine trifluoroacetate salt (**3b**) S7
7-(*N*-(Bis(3'-bromopropyl))aminomethyl)-7-deazaguanine trifluoroacetate salt (**3c**) S7
O^6-methyl preQ$_1$ (**16**, m^{6}preQ$_1$) S8
Supporting Scheme 1 S8

2. HPLC analysis of preQ$_1$ (1) **S9**

3. NMR spectra of compounds 1, 2, 7 and 8 **S10**

4. NMR spectra of compounds 4a–e, 3a–c, 9–13 **S14**

5. ^{1}H-NMR spectrum of compound 16 **S27**

6. References **S27**

1. Synthetic procedures

General. Chemical reagents and solvents were purchased from commercial suppliers (Sigma-Aldrich, Biosynth, abcr) and used without further purification. Dry solvents were used for all non-aqueous reactions, which were carried out under argon atmosphere. Analytical thin-layer chromatography (TLC) was performed on Marchery-Nagel Polygram SIL G/UV254 plates. Silica gel 60 (70–230 mesh) was used for flash column chromatography. Reversed-phase chromatography was performed on an *ÄKTAprime plus* instrumentation using a prepacked *Götech LiChroprep® RP-18* (40–63 µm) column. ^{1}H, and ^{13}C NMR spectra were recorded on *Bruker DRX 300 MHz*, *Bruker Avance 4 Neo 400 MHz*, and *Bruker Avance 4 Neo 700 MHz* instruments. Chemical shifts (δ) are reported relative to tetramethylsilane (TMS) and referenced to the residual proton or carbon signal of the deuterated solvent: DMSO-d_6 (2.50 ppm), Methanol-d_4 (3.31) for ^{1}H NMR; DMSO-d_6 (39.52 ppm), Methanol-d_4 (49.00) for ^{13}C NMR spectra. ^{1}H and ^{13}C assignments are based on COSY, HSQC, and HMBC experiments. ESI-MS experiments were performed on a Thermo Fisher Qexactive Classic. Samples were analyzed in the positive-ion mode.

2-Chloro-3-oxopropanenitrile (6). Sodium methoxide (15.7 g, 291 mmol, 1.1 equiv) was suspended in tetrahydrofuran (500 mL) and cooled to 0 °C. Methyl formate (18.0 mL, 291 mmol, 1.1 equiv) was slowly introduced. Subsequently, 2-chloroacetonitrile (16.7 mL, 264 mmol) was added dropwise over the course of half an hour. After complete addition, the suspension was stirred 3 hours at 0 °C. The reaction was quenched by adjusting the pH to 5 by the addition of concentrated hydrochloric acid at 0 °C. The solids were filtered, and the filtrate was dried over magnesium sulfate. After evaporation **6** (18.3 g, 67%) was obtained as a red oil, that was used without further purification.

preQ$_0$ (7). Sodium acetate (6.14 g, 74.9 mmol, 2.0 equiv) and 2,6-diaminopyrimidine-4(3*H*)one (4.72 g, 37.4 mmol) were vigorously stirred in water (160 mL) at 50 °C for 20 min. **6** (4.65 g, 44.9 mmol, 1.2 equiv, mixed with 2 mL acetonitrile) was added and the reaction was continued for 16 h at this temperature. The reaction was brought to reflux and allowed to cool to room temperature after 1 h. Filtration provided a tan solid, that was washed with small amounts of cold water, followed by acetone and dried in vacuo to give **7** (3.86 g, 59%) as a tan solid. ESI-MS (m/z): $[M+H]^+$ found: 176.0566; $[M+H]^+$ calculated: 176.0567. ^{1}H-NMR (400 MHz, DMSO-d_6 , 25 °C): δ 11.71 (bs, 1H, *H*N(9)), 11.03 (bs, 1H, *H*N(1)), 7.59 (s, 1H, *H*C(8)), 6.41 (bs, 2H, H_2N(2)) ppm. ^{13}C-NMR (100 MHz, DMSO-d_6, 25 °C): δ 157.6 *C*(6), 153.8 *C*(2), 151.7 *C*(4), 127.8 *C*(8), 115.9, 98.8, 85.6 *C*(4) & *C*(5) & *C*(CN) ppm.

preQ$_1$ dihydrochloride (1). A *Büchiglas miniclave steel* hydrogenation apparatus was charged with **7** (150 mg, 0.861 mmol), palladium on carbon (30 mg, 10% catalyst loading) and a mixture of aqueous hydrochloric acid and methanol (1 M, HCl_{aq}/MeOH, 1:1 v/v, 5 mL). The reactor was sealed, hydrogen (30 bar) was applied and the mixture was agitated for 16 h. Afterwards, the solids were filtered and the filtrate was evaporated to give compound **1** (212 mg, 98%) as a beige solid. ESI-MS (m/z): $[M+H]^+$ found: 180.0885; $[M+H]^+$ calculated: 180.0880. ^{1}H-NMR (400 MHz, DMSO-d_6, 25 °C): δ 11.20 (bs, 1H, *H*N(9)), 10.86 (bs, 1H, *H*N(1)), 8.19 (bs, 3H, H_3N^+), 6.78 (s, 1H, *H*C(8)), 6.35 (bs, 2H, H_2N(2)), 4.03 (m, 2H,

H_2CC(7)) ppm. ^{13}C-NMR (100 MHz, DMSO-d_6, 25 °C): δ 158.6 *C*(6), 152.3 *C*(4), 143.1 *C*(4), 117.9 *C*(8), 111.9 *C*(7), 99.0 *C*(5), 35.0 *C*H_2C(7) ppm.

DPQ$_0$ (8). Sodium acetate (3.65 g, 44.4 mmol, 2.0 equiv) and 2,4,6-triaminopyrimidine (2.78 g, 22.2 mmol) were dissolved in water (80 mL) and heated to 50 °C. **6** (2.30 g, 22.2 mmol, 1.0 equiv, mixed with 3 mL acetonitrile) was slowly added to the reaction over the course of 30 min. After complete addition, the mixture was heated to reflux for 16 h. The suspension was allowed to cool to room temperature and the precipitate was filtered and washed sequentially with cold water and small amounts of ice-cold aqueous acetone (acetone/ water, 1:1). Drying in vacuo gave **8** (1.84 g, 47%) as a brown solid. ESI-MS (m/z): $[M+H]^+$ found: 175.0714; $[M+H]^+$ calculated: 175.0727. ^{1}H-NMR (400 MHz, DMSO-d_6): δ 11.82 (bs, 1H, *H*N(9)), 7.69 (s, 1H, *H*C(8)), 6.19 (bs, 2H, H_2N(6)), 5.89 (bs, H_2N(2)) ppm. ^{13}C-NMR (100 MHz, DMSO-d_6): δ 161.5 *C*(6), 157.7 *C*(2), 154.1 *C*(4), 129.3 *C*(8), 117.3 *C*N, 94.5 *C*(5), 82.5 *C*(7) ppm.

DPQ$_1$ dihydrochloride (2). A *Büchiglas miniclave steel* hydrogenation apparatus was charged with **8** (150 mg, 0.861 mmol), palladium on carbon (30 mg, 10% catalyst loading) a mixture of aqueous hydrochloric acid and methanol (1 M, HCl/MeOH, 1:1 v/v, 5 mL). Hydrogen (30 bar) was applied and the mixture was stirred for 16 h. The solids were filtered, and the filtrate was purified by reversed-phase chromatography (eluent A: 0.1% aqueous trifluoroacetic acid; eluent B: acetonitrile, linear gradient 0–17 in 350 mL; flow: 3 mL min^{-1}). After evaporation of the volatiles the remaining solid was coevaporated with dilute hydrochloric acid (1 M, 2 mL) to give dihydrochloride **2** (131 mg, 77%) as a beige solid. ESI-MS: $[M+H]^+$ found 179.1040; $[M+H]^+$ calculated: 179.1040. ^{1}H-NMR (400 MHz, DMSO-d_6, 25 °C): δ 11.95 (s, 1*H*, HN(9)), 8.29 (bs, 2H, H_2N(6)), 8.21 (bs, 3H, H_3N^+), 7.34 (bs, 2H, H_2N(2)), 7.09 (d, J_{HH} = 2.4 Hz, 1H, *H*C(8)), 4.19 (m, 2H, H_2CC(7)) ppm. ^{13}C NMR (150 MHz, D_2O, 25 °C): δ 154.1 & 152.5 & 149.9 *C*(2) & *C*(4) & C(6), 123.5 *C*(8), 109.0 & 94.4 *C*(5) & *C*(7), 35.5 *C*H_2C(7) ppm.

7-Formyl-7-deaza-2,6-diaminopurine (9). Damp Raney-Nickel (1.33 g) was suspended in formic acid (50 mL). **8** (1.00 g, 5.74 mmol) was added. The suspension was stirred for 16 h at room temperature under a hydrogen atmosphere (1 atm). Insolubles were removed by filtration over *Celite* and the filtrate was concentrated in vacuo. The remaining residue was coevaporated twice with methanol, dissolved in hot water (20 mL) and treated with activated charcoal. After filtration over Celite the volume of the solution was reduced to approximately 10 mL. Neutralization with concentrated aqueous ammonia precipitated a light brown solid, which was filtered, washed with cold water and dried in vacuo to give **9** (0.674 g, 66%). TLC: 15 % MeOH in CH_2Cl_2 (containing 1% NH_4OH conc.); R_f 0.43. ESI-MS (m/z): $[M+H]^+$ found: 178.0704; $[M+H]^+$ calculated: 178.0723. ^{1}H-NMR (400 MHz, DMSO-d_6, 25 °C): δ 11.90 (bs, 1H, *H*N(9)), 9.52 (s, 1H, *H*CO), 7.85 (s, 2H, *H*C(8)), 7.38 & 6.90 (s, broad, 2H, H_aN(6) & H_bN(6)), 5.90 (bs, 1H, H_2N(2)) ppm. ^{13}C-NMR (100 MHz, DMSO-d_6, 25 °C): δ 185.4 *C*HO, 161.6 *C*(2), 158.4 *C*(6), 156.0 *C*(4), 135.0 *C*(8), 118.7 *C*(7), 92.14 *C*(5) ppm.

7-Formyl-7-deazaguanine (10). Compound **7** (2.00 g, 11.4 mmol, 1 equiv) was dissolved in formic acid (100 mL) and damp Raney-Nickel (3.5 g) was added. The mixture was stirred at room temperature under hydrogen atmosphere (1 atm) for 20 h. The mixture was filtered through a *Celite* pad. After

removing the volatiles under reduced pressure, the residue was dissolved in a minimal amount of aqueous sodium hydroxide (6 M). The solution was adjusted to neutral pH by the addition of concentrated hydrochloric acid, thereby precipitating the product, which was filtered off, washed with small amounts of cold water and acetone and dried in vacuo to give **10** (1.91 g, 94%) of a red-brown solid. TLC: *n*-butanol : acetic acid : water (2:1:1); R_f 0.41. ESI-MS (m/z): $[M+H]^+$ found: 179.0563; $[M+H]^+$ calculated: 179.0654. ^{1}H-NMR (400 MHz, DMSO-d_6, 25 °C): δ 11.97 (bs, 1H, *H*N(9)), 10.71 (bs, 1H, *H*N(1)), 10.06 (s, 1H, *H*CO), 7.51 (s, 1H, *H*C(8)), 6.37 (s, 2H, H_2N) ppm. ^{13}C-NMR (100 MHz, DMSO-d_6, 25 °C): δ 185.1 *C*HO, 158.6 *C*(6), 153.5 & 153.0 *C*(2) & *C*(4), 124.2 *C*(8), 119.9 *C*(7), 97.9 C(5) ppm.

7-(*N*-(3'-Chloropropyl)aminomethyl)-7-deaza-2,6-diaminopurine (4a). Compound **9** (50.0 mg, 282 µmol, 1 equiv) was suspended in methanol (1.5 mL). Magnesium sulfate (340 mg, 2.82 mmol, 10 equiv), anhydrous potassium carbonate (390 mg, 2.82 mmol, 10 equiv) and 3-chloropropylamine hydrochloride (367 mg, 2.82 mmol, 10 equiv) were added. After 30 minutes of sonication the reaction was allowed to proceed for 16 h at room temperature. The suspension was then cooled to 0 °C and sodium borohydride (96.1 mg, 2.54 mmol, 9 equiv) was added in portions over the course of 30 min and the reaction was stirred at room temperature for another 2.5 hours. The volatiles were removed under reduced pressure and the residue was dissolved in aqueous hydrochloric acid (10%). The solution containing the crude product was purified by reversed-phase chromatography (eluent A: aqueous hydrochloric acid (0.1%); eluent B: acetonitrile, linear gradient: 0–20% B in 350 mL, flow: 3 mL min^{-1}) to give **4a** (15 mg, 18%) as a white solid. TLC: 15 % MeOH in CH_2Cl_2; R_f : 0.46 (free-base). ESI-MS (m/z): $[M+H]^+$ found: 255.1116; $[M+H]^+$ calculated: 255.1119. ^{1}H-NMR (100 MHz, methanol-d_4, 25 °C): δ 7.30 (s, 1H, *H*C(8)), 4.50 (s, 2H, H_2CC(7)), 3.75 (t, J_{HH} = 6.1 Hz, 2H, H_2C(3')), 3.33 (m, 2H, H_2C(1')), 2.26 (m, 2H, H_2C(2')) ppm. ^{13}C-NMR (150 MHz, methanol-d_4, 25 °C): δ 155.0 & 153.1 & 150.8 *C*(2) & *C*(4) & *C*(6), 125.4 *C*(8), 107.8 & 94.9 *C*(5) & *C*(7) 45.9 & 43.7 & *C*(3') & *C*(1'), 42.4 CH_2C(7), 30.1 *C*(2') ppm.

7-(*N*-((2'-Bromoethyl)aminomethyl)-7-deaza-2,6-diaminopurine dihydrobromide (4b). Synthesis was performed according to the *general procedure* (0.282 mmol scale) employing 2-aminoethanol. The crude solid was acidified by 10% hydrobromic acid and purified by reversed-phase chromatography (eluent A: aqueous hydrobromic acid (0.1%); eluent B: acetonitrile, linear gradient: 0–17% B in 350 mL, flow: 3 mL min^{-1}) to give 7-(*N*-((2'-hydroxyethyl)aminomethyl)-7-deaza-2,6-diaminopurine dihydrobromide as an off-white solid. The purified solid was dissolved aqueous hydrobromic acid (65%, 1.5 mL) and heated at 110 °C for 36 h. The volatiles were removed under reduced pressure to give dihydrobromide **4b** as a light-brown solid in quantitative yield (90 mg, 71% yield). TLC: *n*-butanol : acetic acid : water 2 : 1 : 1; R_f : 0.60. ESI-MS (m/z): $[M+H]^+$ found: 285.0457; $[M+H]^+$ calculated: 285.0458. ^{1}H-NMR (400 MHz, DMSO-d_6, 25 °C): δ 11.97 (d, J_{HH} = 2.2 Hz, 1H, *H*N(9)), 8.95 (bs, 2H, H_2N^+), 8.19 (s, 2H, H_2N(6)), 7.30 (bs, 2H, H_2N(2)), (d, J_{HH} = 2.3 Hz, 1H, *H*C(8)), 4.41 (m, 2H, H_2CC(7')), 3.73 (t, 2H, J_{HH} = 7.0 Hz, H_2C(2')), 3.43 (m, 2H, H_2C(1')) ppm. ^{13}C-NMR (100 MHz, DMSO-d_6, 25 °C): δ 153.0 & 151.2 *C*(2) & *C*(4) & *C*(6), 124.2 *C*(7), 106.2 *C*(8), 93.5 *C*(5), 47.0 *C*(1'), 41.5 H_2CC(7), 26.4 *C*(2') ppm.

7-(*N*-(3'-Bromopropyl)aminomethyl)-7-deaza-2,6-diaminopurine (4c). Synthesis was performed according to the *general procedure* (0.282 mmol scale) employing 3-aminopropanol. The crude solid

was acidified by 10% hydrobromic acid and purified by reversed-phase chromatography (eluent A: aqueous hydrobromic acid (0.1%); eluent B: acetonitrile, linear gradient: 0-17% B in 350 mL, flow: 3 mL min^{-1}) to give an off-white solid. The solid was subsequently dissolved in aqueous hydrobromic acid (1.5 mL 65 %) and stirred at 80 °C for 48 h. The solvent was removed under reduced pressure to give dihydrobromide **4c** (101 mg, 78%) as a beige solid. TLC: R_f : 0.68 (free-base). ESI-MS (m/z): $[M+H]^+$ found: 300.0451; $[M+H]^+$ calculated: 300.0454. ^{1}H-NMR (400 MHz, DMSO-d_6, 25 °C): δ 11.95 (s, 1H, *H*N(9)) 8.71 (bs, 1H, H_2N^+), 8.17 (bs, 1H, H_2N(6)), 7.30 (bs, 2H, H_2N(2)), 7.21 (d, J_{HH} = 2.2 Hz, 1H, *H*C(8)), 4.36 (t, J_{HH} = 5.2 Hz, 2H, H_2CC(7))), 3.62 (t, J_{HH} = 6.4 Hz, 2H, H_2C(3')), 3.08 (m, 2H, H_2C(1')), 1.76 (m, 2H, H_2C(2')) ppm. ^{13}C-NMR (150 MHz, methanol-d_4, 25 °C): δ 154.9 & 152.9 & 150.1 *C*(2) & *C*(4) & *C*(6),125.6 *C*(8), 107.8 & 95.0 *C*(5) & *C*(7), 47.0 *C*(1‘), 43.7 *C*H_2C(7), 30.2 & 30.0 *C*(2‘) & *C*(3‘) ppm.

7-(*N*-(4'-Bromobutyl)aminomethyl)-7-deaza-2,6-diaminopurine (4d). Synthesis was performed according to general procedure (0.282 mmol scale) employing 4-aminobutanol. Workup was performed as described for **4c**; **4d** (116 mg, 87%) was obtained as a beige solid. TLC: 15 % MeOH in CH_2Cl_2, R_f: 0.18 (free-base). ESI-MS (m/z): $[M+H]^+$ found: 313.0767; $[M+H]^+$ calculated: 313.0771. ^{1}H-NMR (400 MHz, DMSO-d_6, 25 °C): δ 11.96 (d, J_{HH} = 1.8 Hz, 1H, *H*N(9)), 8.69 (bs, 2H, H_2N^+), 8.19 (s, 2H, H_2N(6)), 7.23 (bs, 2H, H_2N(2)), 7.20 (d, J_{HH} = 2.3 Hz, 1H, *H*C(8)), 4.35 (t, J_{HH} = 5.2 Hz, 2H, H_2CC(7)), 3.56 (t, J_{HH} = 6.3 Hz, 2H, H_2C(1')), 1.91-1.70 (m, 4H, H_2C(2') & H_2C(3')) ppm. ^{13}C-NMR (100 MHz, methanol-d_4, 25 °C): δ 155.1 & 153.1 & 150.5 *C*(2) & *C*(4) & *C*(6), 125.3 *C*(8), 107.8 & 95.0 *C*(5) & *C*(7), 47.5 *C*(1'), 43.6 *C*H_2C(7), 33.3 *C*(4'), 30.8 *C*(3'), 26.0 *C*(2') ppm.

7-(*N*-(3'-Hydroxypropyl)aminomethyl)-7-deaza-2,6-diaminopurine trifluoroacetate salt (11). Synthesis was performed according to *general procedure* (0.452 mmol scale) employing 3-aminopropanol. The crude solid was acidified by trifluoroacaetic acid (10%) and purified by reversed-phase chromatography (eluent A: aqueous trifluoroacetic acid (0.1%); eluent B: acetonitrile, linear gradient: 0-15% B in 350 mL, flow: 3 mL min^{-1}) to give **11** (159 mg, 76%) as an off-white crystalline solid. TLC: 15 % MeOH in CH_2Cl_2, R_f : 0.16 (free-base). ESI-MS (m/z): $[M+H]^+$ found: 237.1454; $[M+H]^+$ calculated: 237.1458. ^{1}H-NMR (400 MHz, DMSO-d_6, 25 °C): δ 11.99 (bs, 1H, *H*N(9)), 8.76 (bs, 2H, H_2N^+), 8.27 (bs, 2H, H_2N(6)), 7.32 (bs, 2H, H_2N(6)), 7.20 (d, J_{HH} = 2.4 Hz, 1H, *H*C(8)), 4.31 (t, J_{HH} = 5.3 Hz, 2H, H_2CC(7)) 3.48 (t, J_{HH} = 5.9 Hz, 2H, H_2C(3')), 3.00 (m, 2H, H_2C(1')) 1.78 (m, 2H, H_2C(2')) ppm. ^{13}C-NMR (150 MHz, DMSO-d_6, 25 °C): δ 151.2 *C*(2) or *C*(4) or *C*(6), 123.5 *C*(8), 93.3 *C*(5), 58.0 *C*(3'), 44.0 *C*(1'), 41.4 *C*H_2C(7), 28.3 *C*(2') ppm.

7-(*N*-(3'-Iodopropyl)aminomethyl)-7-deaza-2,6-diaminopurine trifluoroacetate salt (4e). Compound **11** (40.0 mg, 0.11 mmol, 1.0 equiv), 1*H*-imidazole (8.6 mg, 0.13 mmol, 1.2 equiv) and triphenylphosphine (33.1 mg, 0.126 mmol, 1.15 equiv) were dissolved in DMF (170 µL). Iodine (32.1 mg, 0.126 mmol, 1.15 equiv in 35 µL DMF) was added. After 3.5 h at room temperature the solution was concentrated under reduced pressure and the oily residue was suspended in aqueous trifluoroacetic acid (1 mL, 1%). The insoluble were removed by centrifugation and the supernatant was purified by reversed-phase chromatography (eluent A: aqueous trifluoroacetic acid (0.1%), eluent B:

acetonitrile, linear gradient: 0–15% B in 350 mL, flow: 3 mL min^{-1}) to give **4e** (50.0 mg, 99%) of a colorless crystalline solid. TLC: 20 % MeOH in CH_2Cl_2 (containing 1% NH_4OH); R_f : 0.62. ESI-MS (m/z): $[M+H]^+$ found: 347.0473; $[M+H]_+$ calculated: 347.0476. ^{1}H-NMR (700 MHz, DMSO-d_6, 25 °C): δ 13.02 (bs, 1H, *H*N(1)), 12.06 (bs, 1H, *H*N(9)), 8.91 (bs, 2H, H_2N^+)), 8.21 (bs, 2H, H_2N(6)), 7.25 (bs, 2H, H_2N(2)), 7.15 (s, 1H, *H*C(8)), 4.33 (s, 2H, H_2*CC*(7)), 3.74 (t, J_{HH} = 6.4 Hz, 2H, H_2C(3')), 3.91 (m, 2H, H_2C(1')), 2.09 (m, 2H, H_2C(2')) ppm. ^{13}C-NMR (176 MHz, DMSO-d_6, 25 °C): δ 159.1 (q, J_{CF} = 32 Hz, CF_3*C*OO^-), 151.9 *C*(4), 123.4 *C*(8), 117.0 (q, J_{CF} = 298 Hz, *C*F_3COO^-), 106.5 *C*(7), 93.5 *C*(5), 43.7 *C*(1'), 42.4 *C*(3'), 41.7 *C*H_2C(7), 28.5 *C*(2') ppm.

7-(*N*-(3'-Bromopropyl)aminomethyl)-7-deazaguanine dihydrobromide (3a). Synthesis was performed according to *general procedure* (0.282 mmol scale) employing 3-aminopropanol. Workup was performed as described for **4b**; **3a** (85 mg, 65%) was obtained as a beige solid. TLC: 20 % MeOH in CH_2Cl_2 (containing 1% NH_4OH); R_f : 0.68. ESI-MS (m/z): $[M+H]^+$ found: 300.0451; $[M+H]^+$ calculated: 300.0454. ^{1}H-NMR (400 MHz, DMSO-d_6, 25 °C): δ 11.36 (s, 1H, *H*N(9)) 11.02 (s, broad, *H*N(1)), 9.00 (bs, 1H, H_2N^+), 6.87 (d, J_{HH} = 2.1 Hz , 1H, *H*C(8)), 4.19 (t, J_{HH} = 5.2 Hz, 2H, H_2CC(7))), 3.60 (t, J_{HH} = 6.5 Hz, 2H, H_2C(3')), 3.05 (dd, J_{HH} = 6.6 and 7.3 Hz 2H, H_2C(1')), 2.17 (dd, J_{HH} = 6.6 and 7.3 Hz, 2H, H_2C(2')) ppm. ^{13}C-NMR (100 MHz, methanol-d_4, 25 °C): δ 159.6 *C*(6), 152.6 *C*(2), 139.6 *C*(4), 121.4 *C*(8), 111.12 & 100.9 *C*(5) & *C*(7), 46.6 *C*(1'), 43.6 *C*H_2C(7), 30.1 & 29.9 *C*(2') & *C*(3') ppm.

7-(*N*-(3'-Hydroxypropyl)aminomethyl)-7-deazaguanine trifluoroacetate salt (12). Synthesis was performed according to *general procedure* (1.12 mmol scale) employing 3-aminopropanol. Workup as described for **11**; **12** (469 mg, 90%) was obtained as a colorless crystalline solid. ESI-MS (m/z): $[M+H]^+$ found: 238.1309; $[M+H]^+$ calculated: 238.1304. ^{1}H-NMR (400 MHz, DMSO-d_6, 25 °C): δ 11.35 (d, J_{HH} = 1.4 Hz, 1H, *H*N(9)), 11.09 (bs, 1H, *H*N(1)), 8.97 (bs, 2H, H_2N^+), 6.82 (d, J_{HH} = 2.2 Hz 1H, *H*C(8)), 6.59 (bs, 2H, H_2N(2)), 4.16 (t, J_{HH} = 5.0 Hz, 2H, H_2CC(7)), 3.47 (t, J_{HH} = 6.0 Hz, 2H, H_2C(3')), 3.00 (m, 2H, H_2C(1')), 1.76 (m, 2H, H_2C(2')) ppm. ^{13}C-NMR (100 MHz, DMSO-d_6, 25 °C): δ 160.3 *C*(6), 158.6 (q, J_{CF} = 35.2 Hz, CF_3*C*OO^-), 152.8 *C*(2), 151.4 *C*(4), 117.5 *C*(8), 116.1 (q, J_{CF} = 293.3 Hz, *C*F_3COO^-), 108.6 *C*(7), 98.3 *C*(5), 58.00 *C*(3'), 43.8 *C*(1'), 42.9 *C*H_2C(7), 28.8 *C*(2') ppm.

7-(*N*-(3'-Hydroxypropyl)-*N*-(tert-butyloxycarbonyl)aminomethyl)-7-deazaguanine (13). To a solution of **12** (132 mg, 285 µmol) in methanol (4 mL) was sequentially added triethylamine (160 µL, 1.14 mmol, 4 equiv) and di-*tert*-butyl dicarbonate (186 mg, 854 µmol, 3 equiv) in dichloromethane (0.5 mL). After stirring for 3 h at room temperature, the reaction was quenched with methanolic ammonia (7 M, 0.5 mL), evaporated to dryness and purified by flash column chromatography (5–15% MeOH in CH_2Cl_2) to provide **13** (83 mg, 86%) as an off-white solid. TLC: 15% MeOH in CH_2Cl_2, R_f: 0.38. ESI-MS (m/z): $[M+H]^+$ found: 338.1818; $[M+H]^+$ calculated: 338.1823. ^{1}H-NMR (400 MHz, DMSO-d_6, 25 °C): δ 10.82 (bs, 1H, *H*N(9), 10.22 (s, 1H, *H*N(1)), 6.36 (bs, 1H, *H*C(8)), 6.04 (s, 2H, H_2N(2)), 4.40 (s, 2H, H_2CC(7)), 4.34 (m, 1H, *H*O(3')), 3.42-3.28 (m, 2H, H_2C(1')), 3.24 (m, 2H, H_2C(3')), 1.59 (m, 2H, H_2C(2')), 1.39 (s, 9 H, H_3C(Boc) ppm. ^{13}C-NMR (100 MHz, DMSO-d_6, 25 °C): δ 159.3 *C*(6), 154.8 & 152.3 *C*(2) & *C*(carbonyl, Boc), 115.2 & 114.3 *C*(7) & *C*(8), 98.5 *C*(5), 78.3 *C*(tertiary, Boc), 58.7 *C*(1'), 43.31 *C*(3'), 41.9 *C*H_2C(7), 31.5 *C*(2') ppm.

7-(*N*-(3'-Mesyloxypropyl)aminomethyl)-7-deazaguanine trifluoroacetate salt (3b). To a solution of **13** (151 mg, 448 µmol) and triethylamine (187 µL, 134 µmol, 3 equiv) in *N,N*-dimethylformamide (2.0 mL) at 0 °C was added methanesulfonyl chloride (42 µL, 537 µmol, 1.2 equiv). After 1.5 h, the volatiles were removed in vacuo and the residue was coevaporated with toluene twice. Swift column chromatography on silica gel (5–8% MeOH in CH_2Cl_2) provided compound as a white foam. TLC: 15% MeOH in CH_2Cl_2; R_f : 0.58. Due to the instability of the Boc-protected compound, purified product **14** was directly dissolved in chloroform (2.0 mL) and treated with trifluoroacetic acid (300 µL). After 1 h at room temperature the volatiles were removed in vacuo and the residual oil was coevaporated with dichloromethane 3 times until a white crystalline solid was obtained. The crude product was recrystallized from acetonitrile (approx. 25 µL per mg of crude material, the hot solution was filtered). Upon cooling to –20 °C, crystals appeared which were collected and dried to give **3b** as a white crystalline solid (109 mg, 45%). TLC: 15% MeOH in CH_2Cl_2; R_f : baseline. ESI-MS (m/z): $[M+H]^+$ found: 316.1072; $[M+H]^+$ calculated: 316.1074. ^{1}H-NMR (400 MHz, DMSO-d_6, 25 °C): δ 11.32 (d, J_{HH} = 2.1 Hz, 1H, *H*N(9)), 10.97 (s, 1H, *H*N(1)), 9.06 (bs, 2H, H_2N^+), 6.83 (d, J_{HH} = 2.1 Hz, 1H, *H*N(9)), 6.43 (bs, 2H, H_2N(2)), 4.30 (t, J_{HH} = 6.1 Hz, 2H, H_2C(3')), 4.20 (t, J_{HH} = 5.1 Hz, H_2CC(7)), 3.20 (s, 3H, H_3C(Oms)), 3.11-3.02 (m, 2H, H_2C(1')), 2.10-2.01 (m, 2H, H_2C(2')) ppm. ^{13}C-NMR (150 MHz, DMSO-d_6, 25 °C): δ 160.32 *C*(6), 158.3 (q, J_{CF} = 36.5 Hz, CF_3COOH), 152.7 *C*(2), 152.0 *C*(4), 119.0 *C*(8), 116.0 (q, J_{CF} = 294.4 Hz, *C*F_3COOH), 108.3 & 98.2 *C*(5) & *C*(7), 67.4 *C*(3'), 42.9 *C*H_2C(7), 42.5 C(1'), 36.6 *C*H_3(Oms), 25.6 *C*(2') ppm.

7-(*N*-(Bis(3'-bromopropyl))aminomethyl)-7-deazaguanine trifluoroacetate salt (3c). preQ_1 dihydrochloride (110 mg, 436 µmol) together with bis(3-hydroxypropyl)amine (581 mg, 4.36 mmol, 10 equiv) was dissolved in tetrahydrofuran/methanol/water (1:1:1, 4 mL) and heated to 80 °C for 24 h. The volatiles were removed in vacuo and the residual oil was heated to 80 °C for 24 h in aqueous hydrobromic acid (65%). The volatiles were removed under reduced pressure and the residual solid purified by reversed-phase chromatography (eluent A: aqueous trifluoroacetic acid (0.1%), eluent B: acetonitrile, linear gradient: 0–25% B in 350 mL, flow: 3 mL min^{-1}) to give **3c** (120 mg, 42%) as an off-white solid. ESI-MS (m/z): $[M+H]^+$ found: 420.0034; $[M+H]^+$ calculated: 420.0029. ^{1}H-NMR (400 MHz, DMSO-d_6, 25 °C): δ 11.42 (d, J_{HH} = 1.6 Hz, 1H, *H*N(9)), 11.02 (s, 1H, *H*N(1)), 10.06 (bs, 1H, HN^+), 6.92 (d, J_{HH} = 1.6 Hz, 1H, *H*C(9)), 6.46 (bs, 2H, H_2N(9)), 4.39 (d, J_{HH} = 4.4 Hz, 1H, H_2CC(7)), 3.59 (t, J_{HH} = 6.4 Hz, 4H, H_2C(3')), 3.33 -3.15 (m, 4H, H_2C(1')), 2.38-2.19 (m, 4H, H_2C(2')) ppm. ^{13}C-NMR (100 MHz, methanol-d_4, 25 °C): δ 162.7 *C*(6), 161.33 (q, J_{CF} = 37.6 Hz, CF_3COOH), 154.4 *C*(2), 152.6 *C*(4), 120.8 *C*(8), 117.2 (q, J_{CF} = 288.3 Hz, *C*F_3COOH), 108.7 & 99.8 *C*(5) & *C*(7), 52.7 *C*H_2C(7), 52.0 *C*(1'), 29.9 *C*(3'), 28.1 *C*(2') ppm.

O^6-Methyl preQ$_1$ (16, m^{6}preQ$_1$)

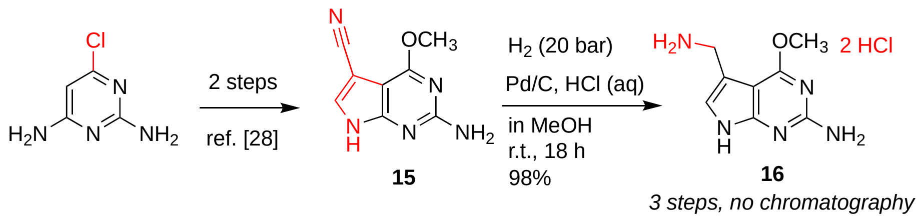

Scheme S1: Using the hydrogenation conditions established in this work, the synthetic target of an earlier study, *O*^{6}-methyl preQ$_1$ (m^{6}preQ$_1$, **16**), can be directly synthesized through reduction of *O*^{6}-methyl preQ$_0$ (**15**), significantly shortening the synthetic route compared to the previously published procedure [1].

***O*^{6}-methyl preQ$_1$ dihydrochloride (m^{6}preQ$_1$, 16).** Compound **15** (prepared according to ref. [1]) (120 mg, 634 µmol) was suspended in methanol/aqueous hydrochloric acid (1 M, 1:1, 4 mL). Palladium on charcoal (19% loading, 20 mg) was added and the reaction was stirred for 18 h in a *Büchiglas miniclave steel* hydrogenation apparatus (30 bar of hydrogen). After filtration over a *Celite* pad the solvents were removed in vacuo to give **16** (165 mg, 98%) as an off-white solid. TLC: 15% MeOH, 1% NEt_3 in CH_2Cl_2, R_f : 0.56. ESI-MS (m/z): $[M+H-NH_3]^+$ calculated: 177.0771; $[M+H-NH_3]^+$ found: 177.0767; $[M+H]^+$ calculated: 194.1036; $[M+H]^+$ found: 194.1032. ^{1}H-NMR (400 MHz, DMSO-d_6, 25 °C): δ 12.34 (bs, 1H, *H*N(9)), 8.44 (bs, 3H, H_3N^+), 7.14 (d, 1H, J_{HH} = 2.0 Hz, *H*C(8)), 4.06 (s, 3H, H_3CO(6)), 4.03 (m, 2H, H_2CC(7)) ppm.

2. HPLC analysis of preQ$_1$ (1)

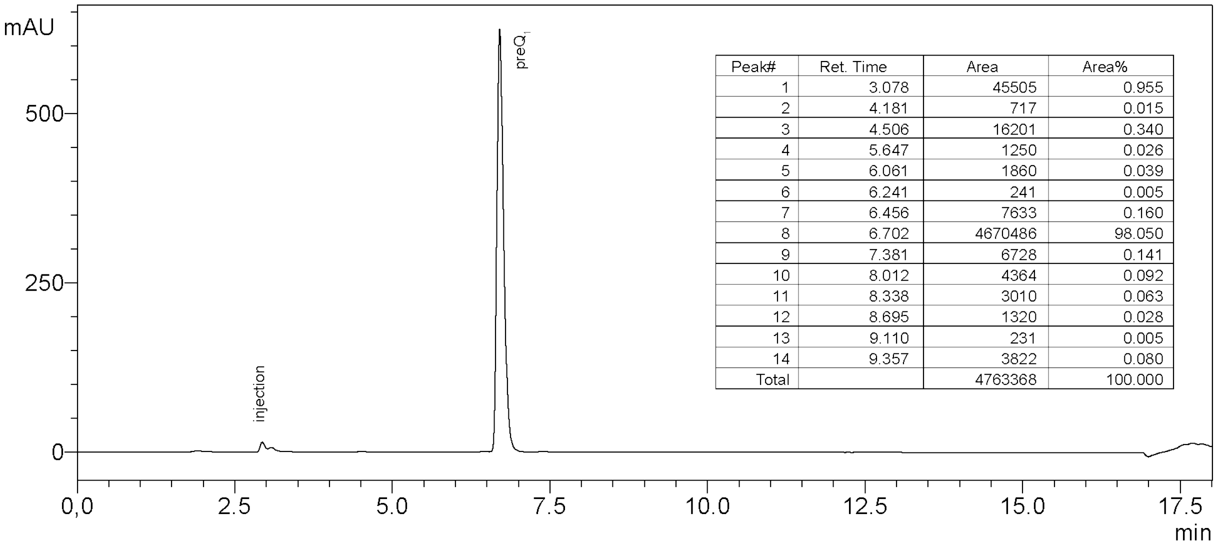

Peak#	Ret. Time	Area	Area%
1	3.078	45505	0.955
2	4.181	717	0.015
3	4.506	16201	0.340
4	5.647	1250	0.026
5	6.061	1860	0.039
6	6.241	241	0.005
7	6.456	7633	0.160
8	6.702	4670486	98.050
9	7.381	6728	0.141
10	8.012	4364	0.092
11	8.338	3010	0.063
12	8.695	1320	0.028
13	9.110	231	0.005
14	9.357	3822	0.080
Total		4763368	100.000

Supporting Figure 1: RP-HPLC analysis of preQ$_1$ · 2 HCl prepared by hydrogenation of preQ$_0$ as described above. The experiment was performed on a *Shimadzu Prominence-i LC-2030 3D Plus* instrumentation equipped with a *Waters XBridge™ C18 5 µm* column (4.6 x 150 mm) at 25 °C. The gradient was linear (0-15% ACN in 0.1% trifluoroacetic acid in 15 min) at a flow rate of 1 mL s^{-1}. UV absorbance was monitored at 280 nm, indicating purity > 98%. Together, HPLC, NMR and mass spectrometry data confirm the purity of the product, eliminating the need for a dedicated purification step.

3. NMR spectra of compounds 1, 2, 7 and 8

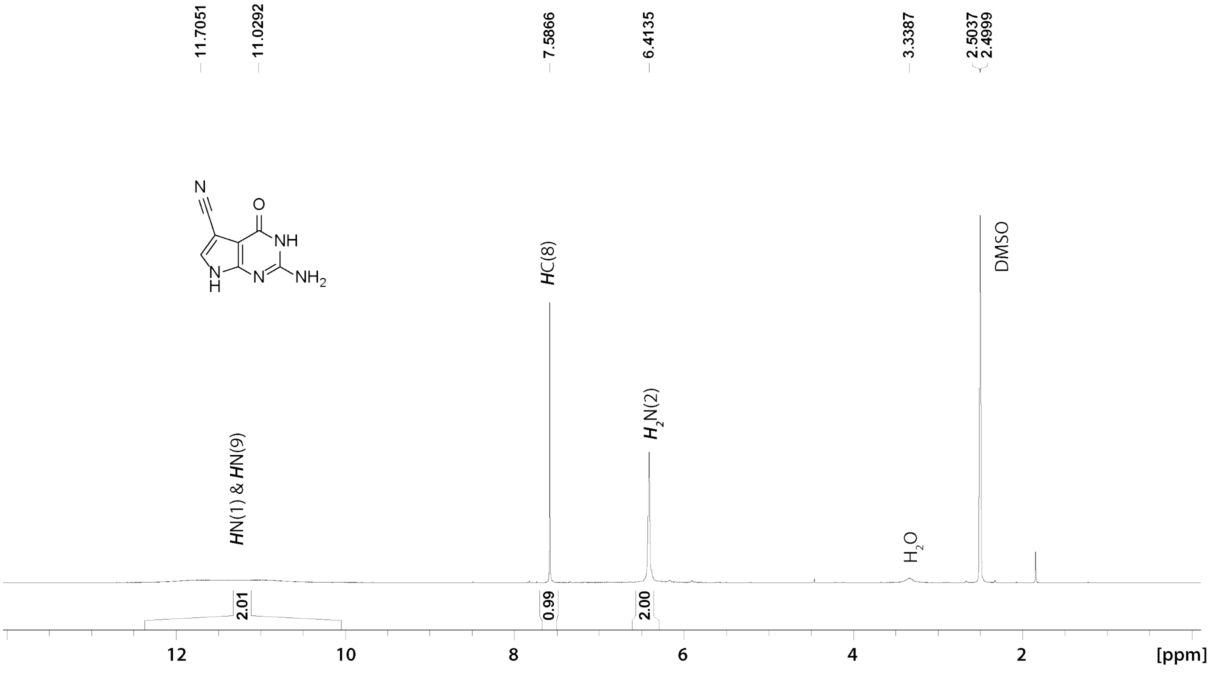

^{1}H-NMR (400 MHz, DMSO-d_6, 25 °C) spectrum of compound **7**.

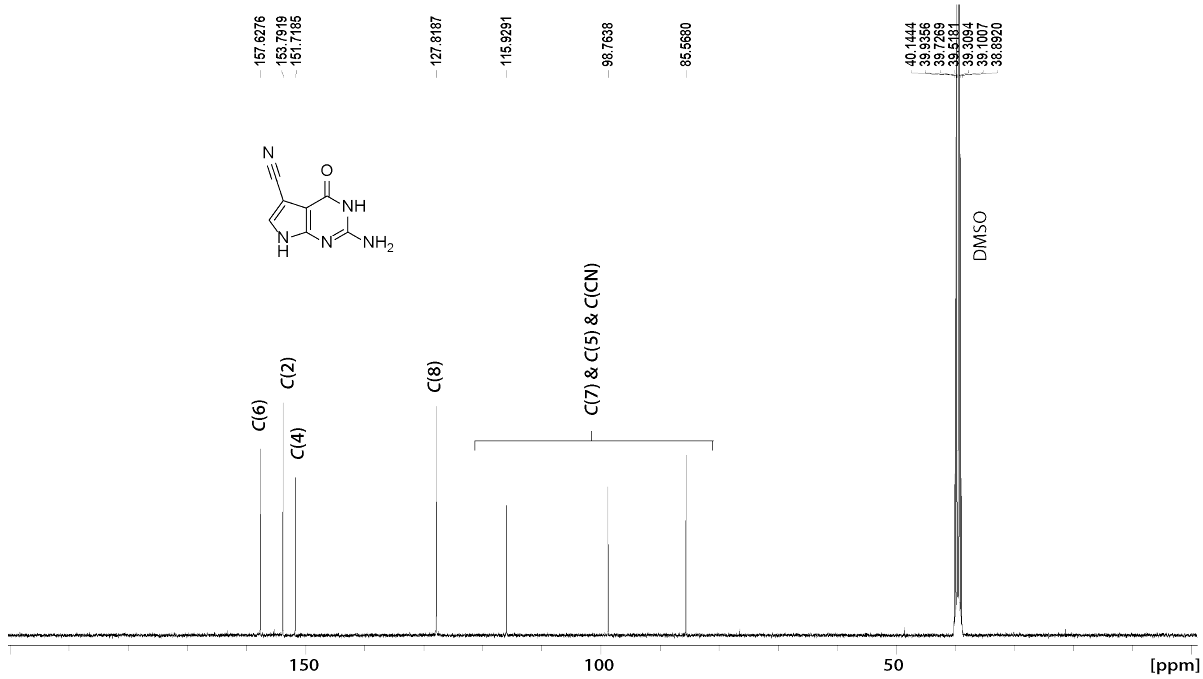

^{1}H-NMR (100 MHz, DMSO-d_6, 25 °C) spectrum of compound **7**.

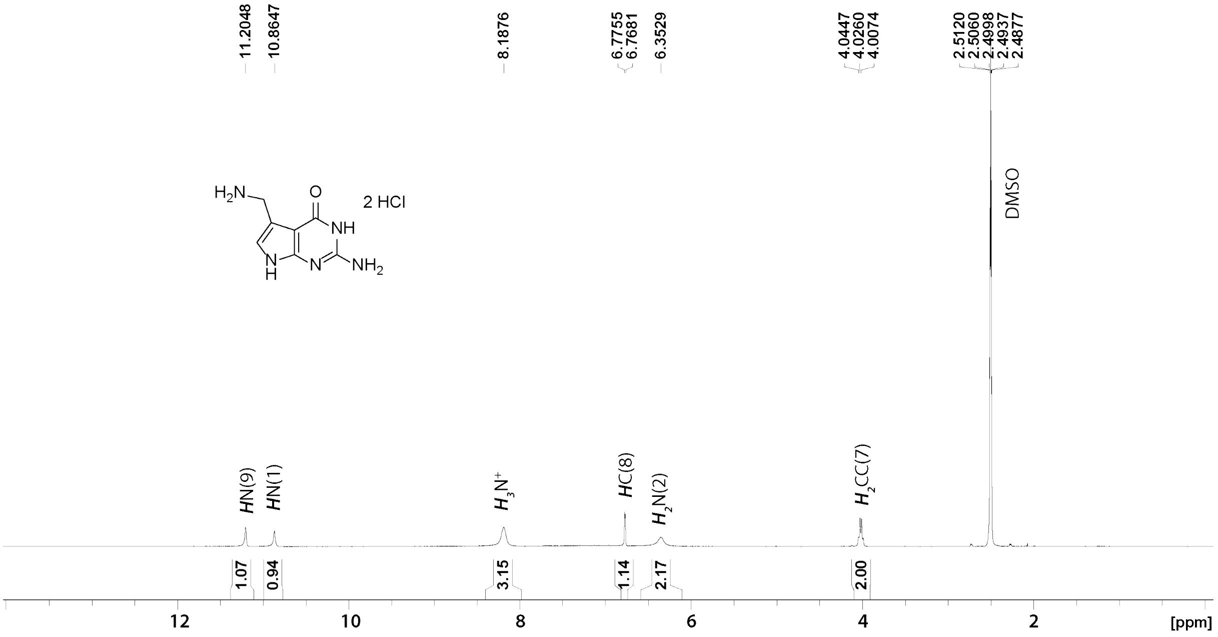

^{1}H-NMR (400 MHz, DMSO-*d*6, 25 °C) spectrum of compound **1**.

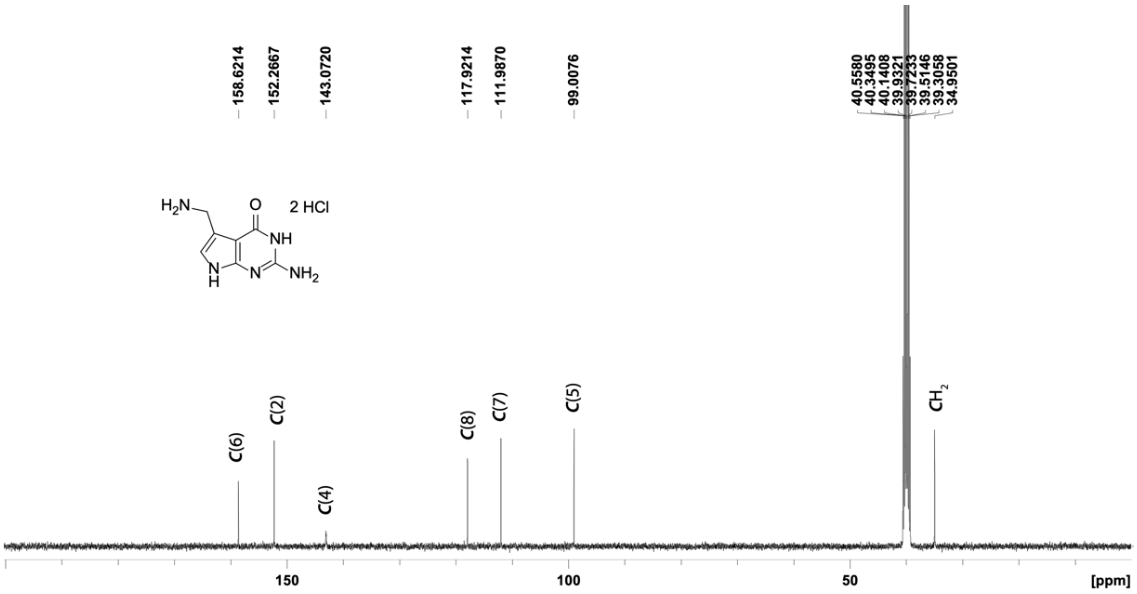

^{13}C-NMR (100 MHz, DMSO-*d*6, 25 °C) spectrum of compound **1**.

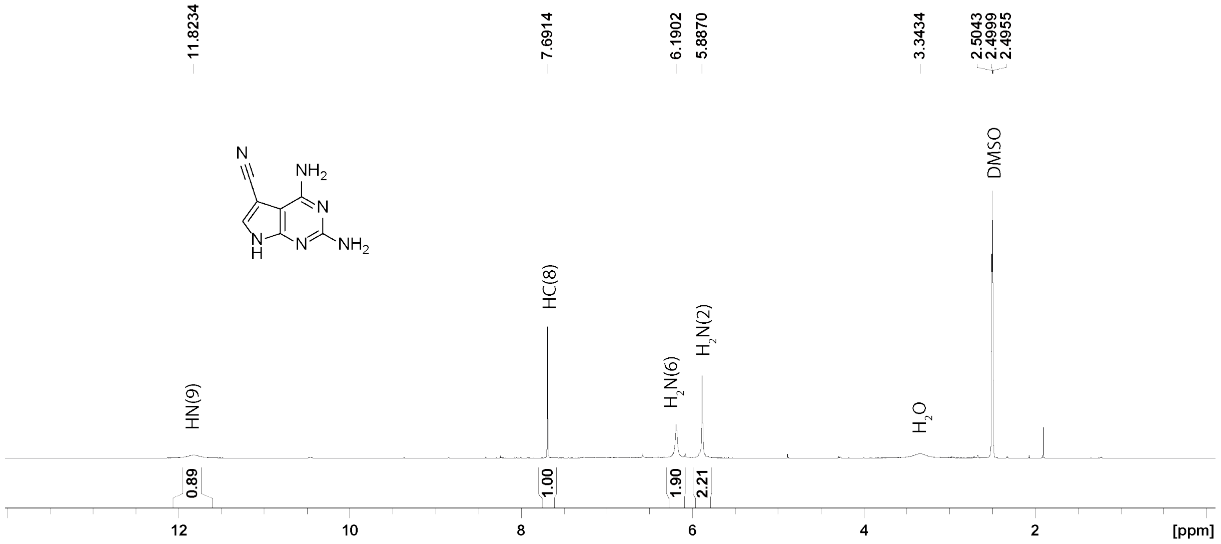

^{1}H-NMR (400 MHz, DMSO-*d*6, 25 °C) spectrum of compound **1**.

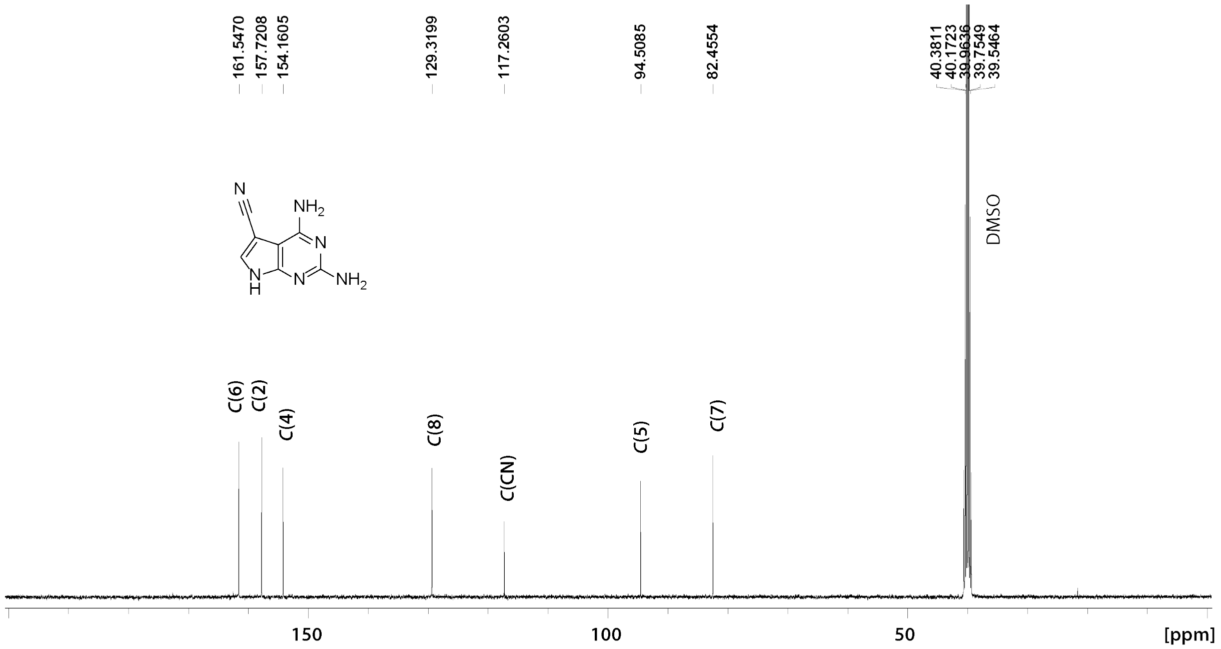

^{13}C-NMR (100 MHz, DMSO-*d*6, 25 °C) spectrum of compound **8**.

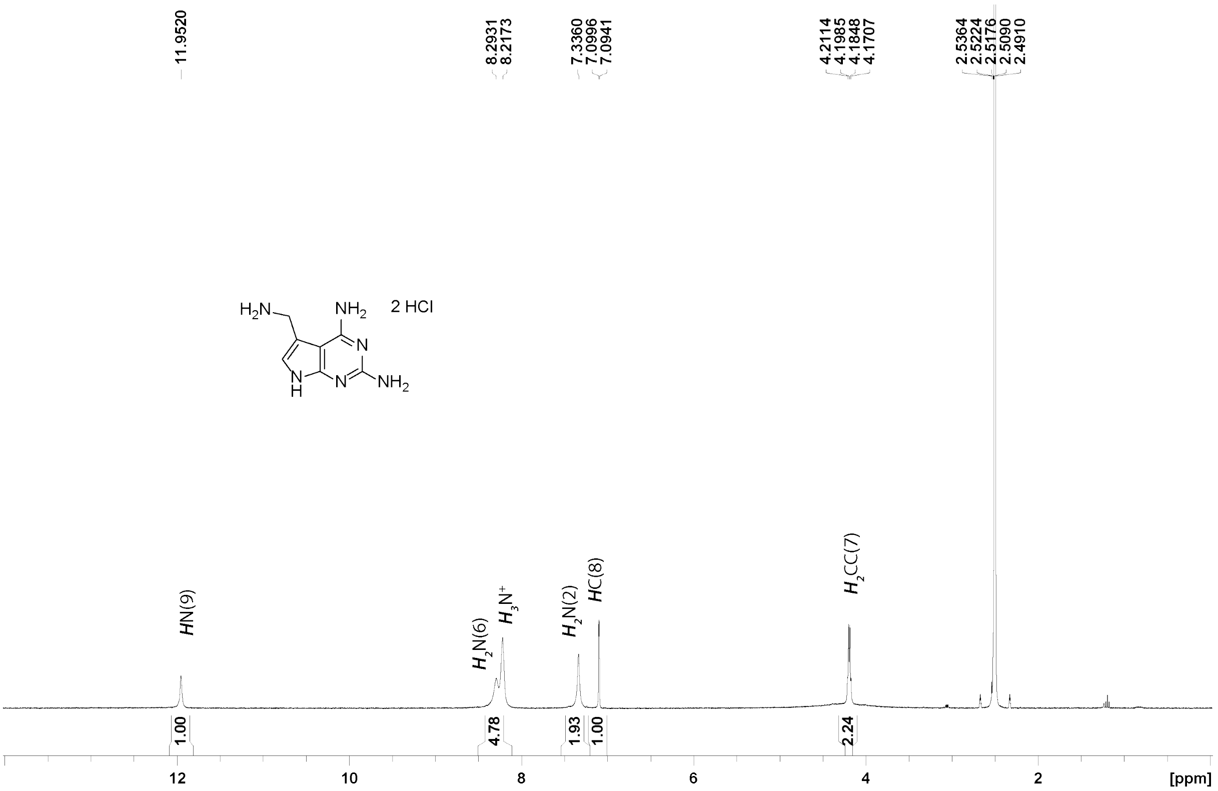

^{1}H-NMR (400 MHz, DMSO-d_6, 25 °C) spectrum of compound **2**

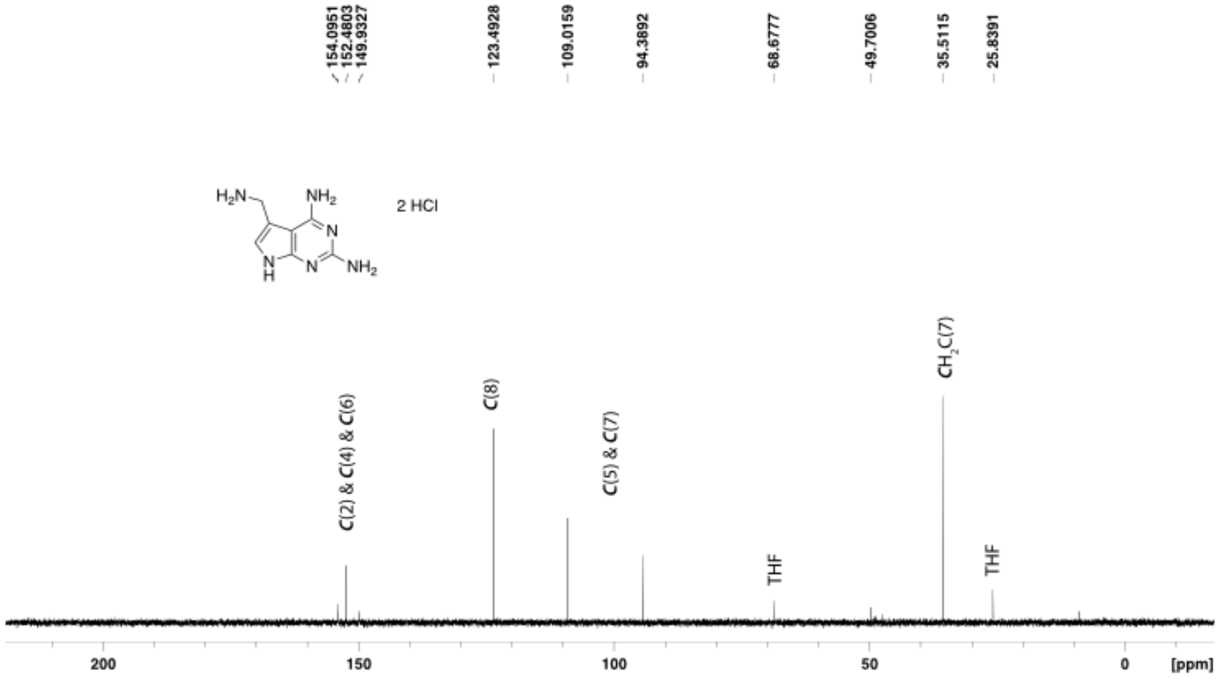

^{13}C-NMR (600 MHz, DMSO-d_6, 25 °C) spectrum of compound **2**.

4. NMR spectra of compounds 4a-e, 3a-c, 9-13

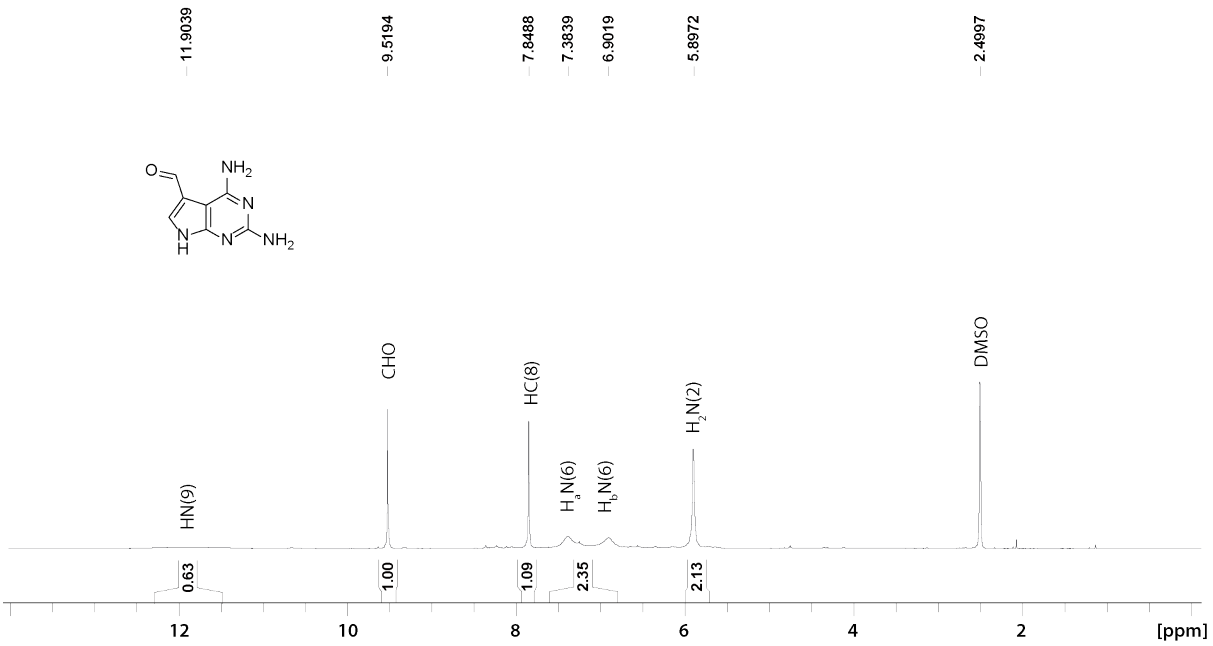

^{1}H-NMR (400 MHz, DMSO-*d6,* 25 °C) spectrum of compound **9**.

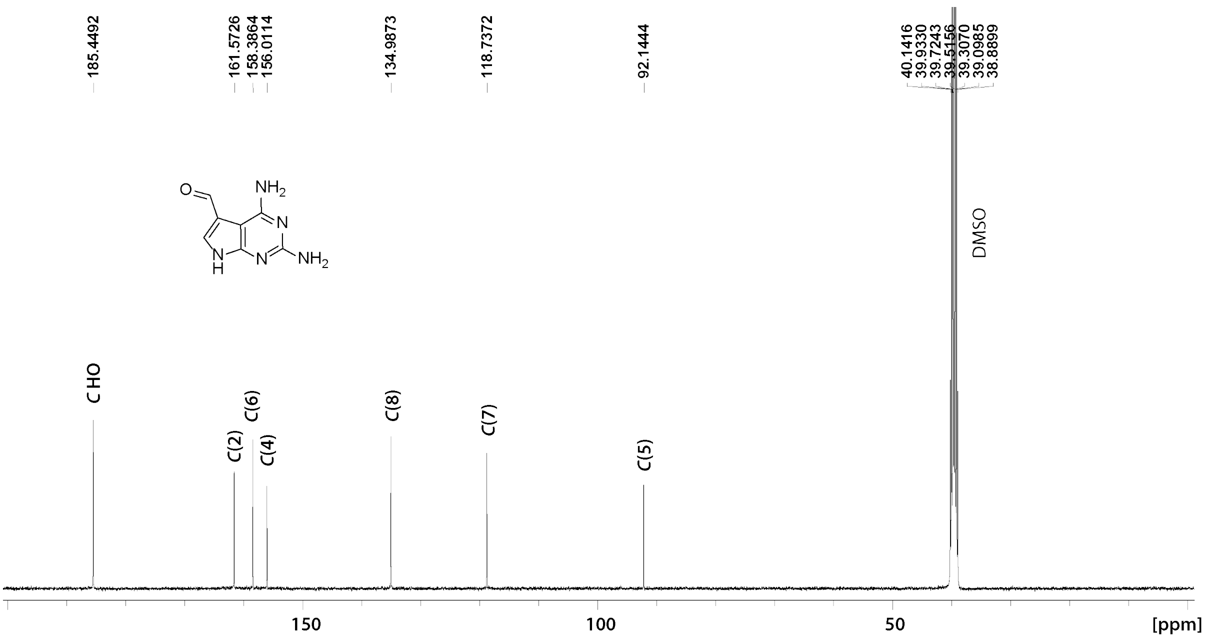

^{13}C-NMR (100 MHz, DMSO-*d6,* 25 °C) spectrum of compound **9**.

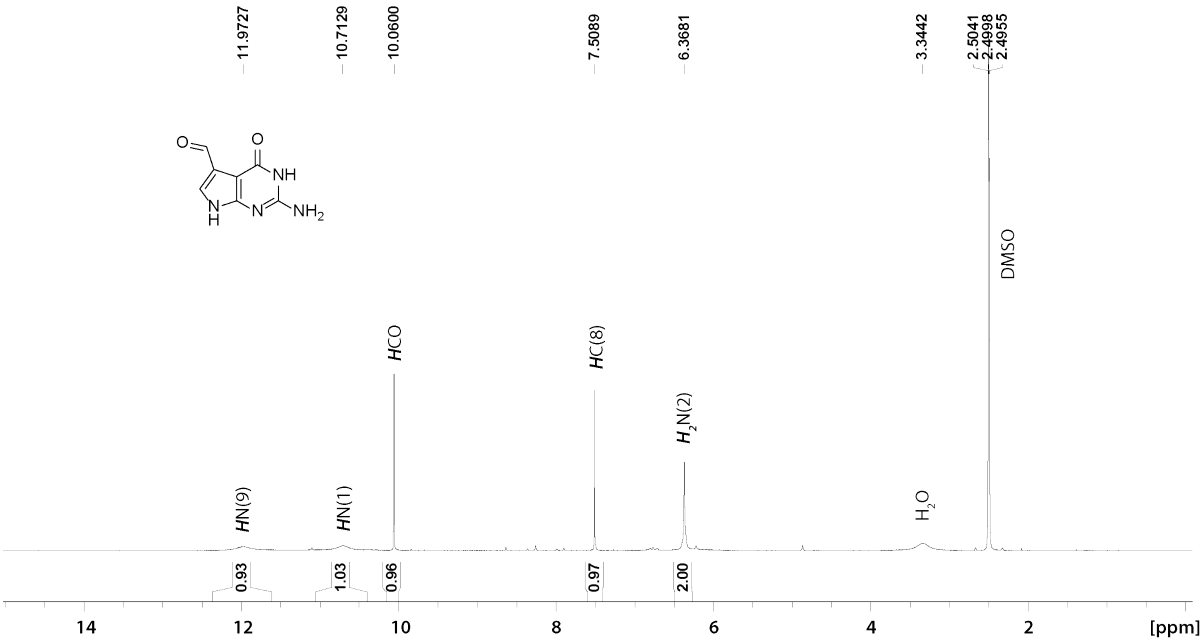

^{1}H-NMR (400 MHz, DMSO-d_6, 25 °C) spectrum of compound **10**.

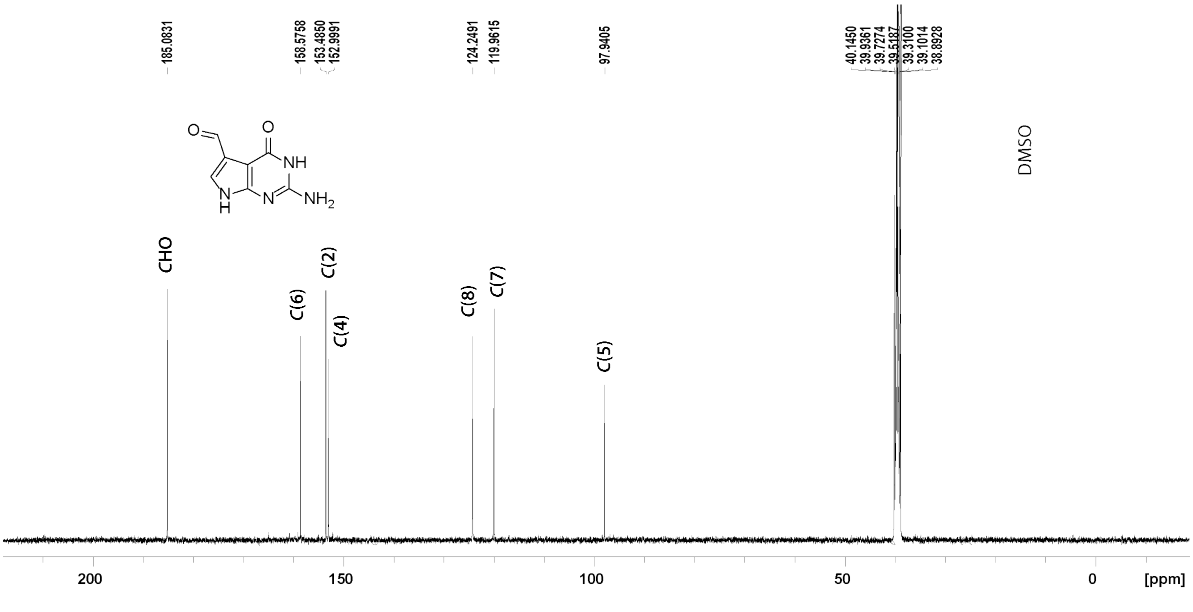

^{13}C-NMR (400 MHz, DMSO-d_6, 25 °C) spectrum of compound **10**.

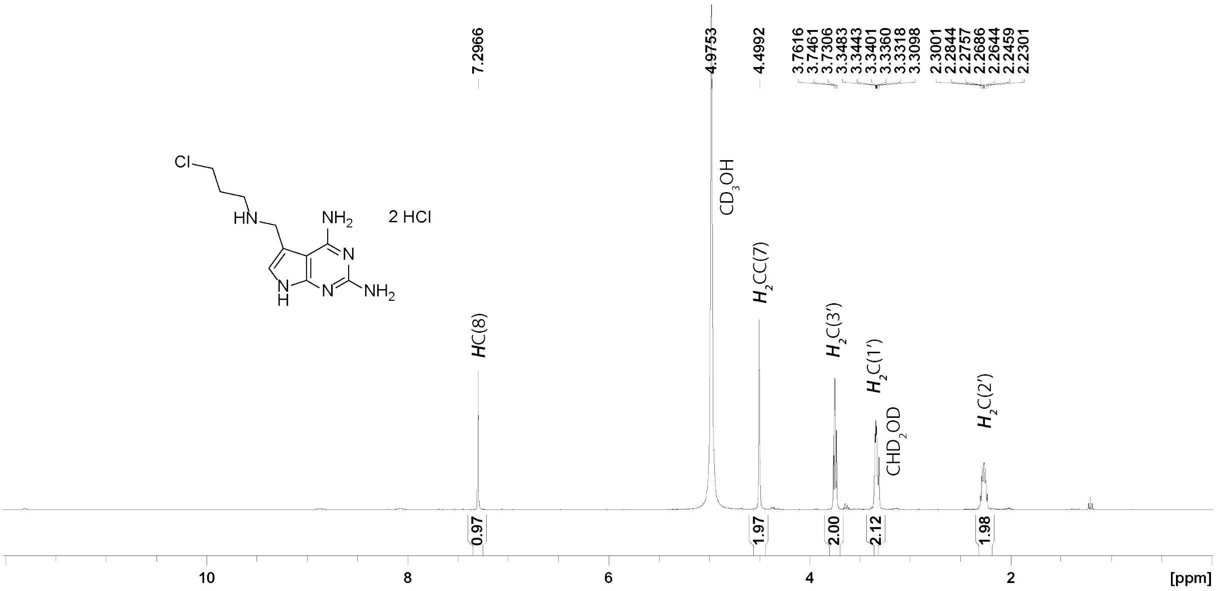

^{1}H-NMR (400 MHz, methanol-*d4,* 25 °C) spectrum of compound **4a**.

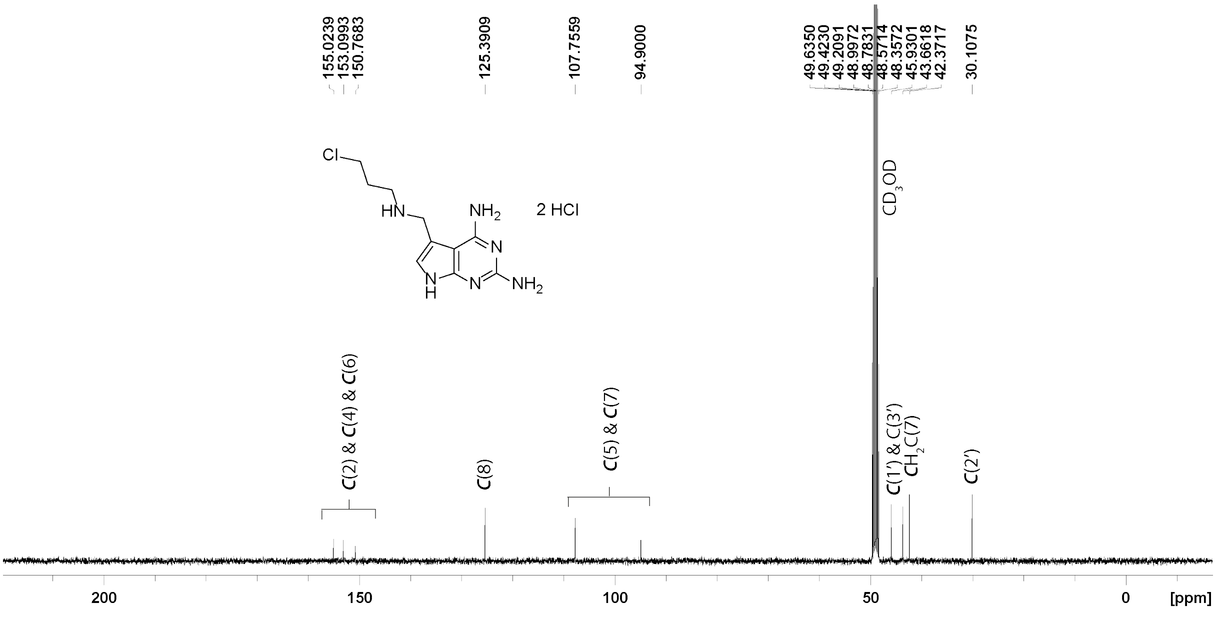

^{13}C-NMR (100 MHz, methanol-*d4,* 25 °C) spectrum of compound **4a.**

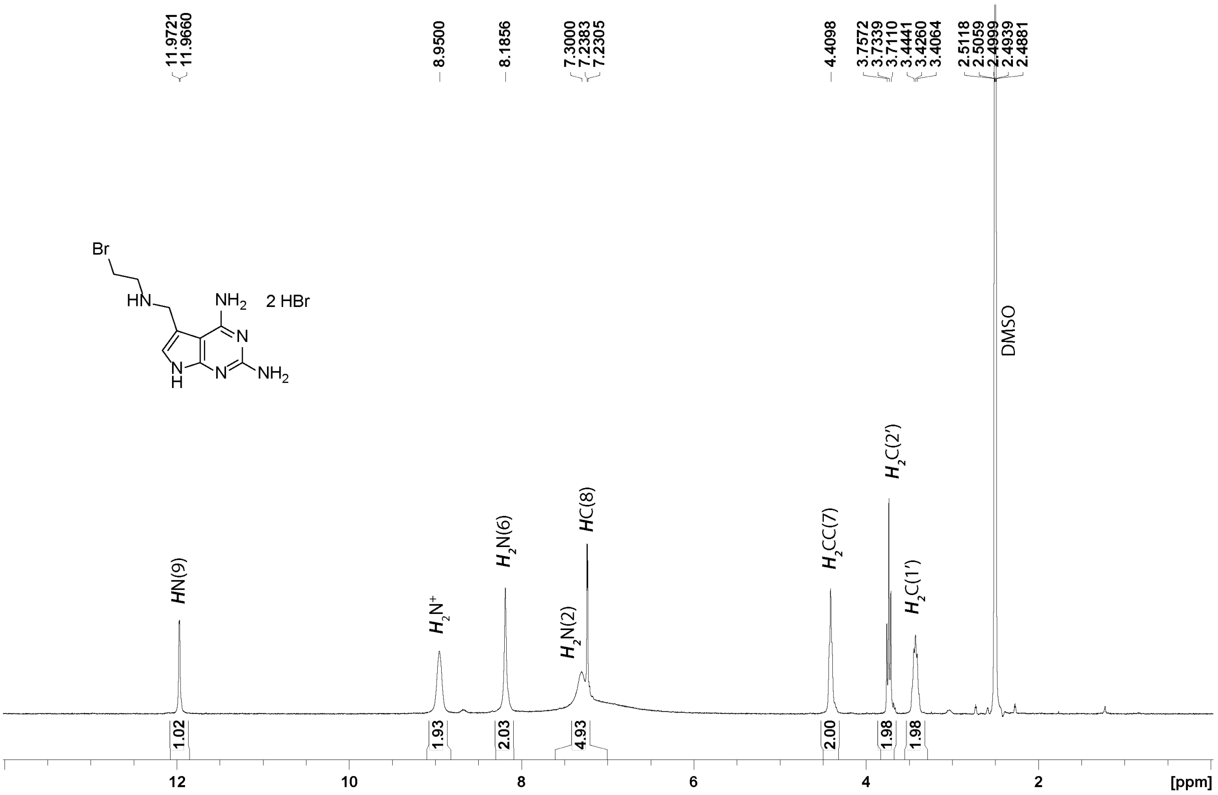

^{1}H-NMR (400 MHz, DMSO-*d6*, 25 °C) spectrum of compound **4b**.

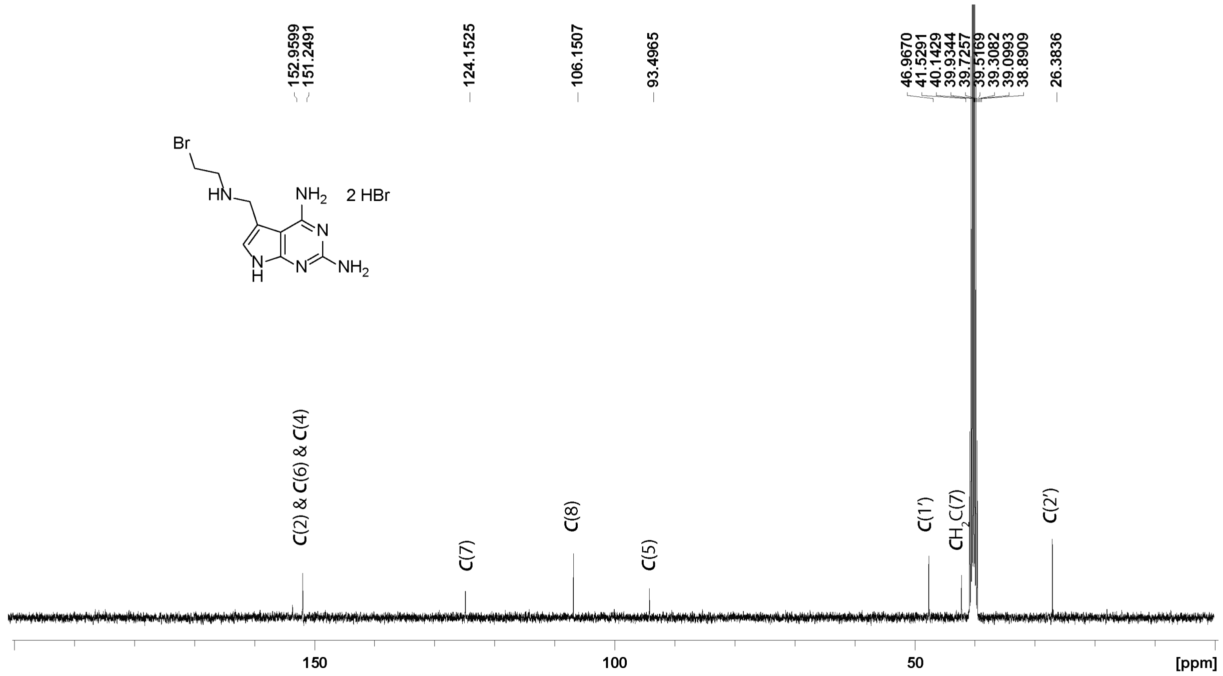

^{13}C-NMR (100 MHz, DMSO-*d6,* 25 °C) spectrum of compound **4b.**

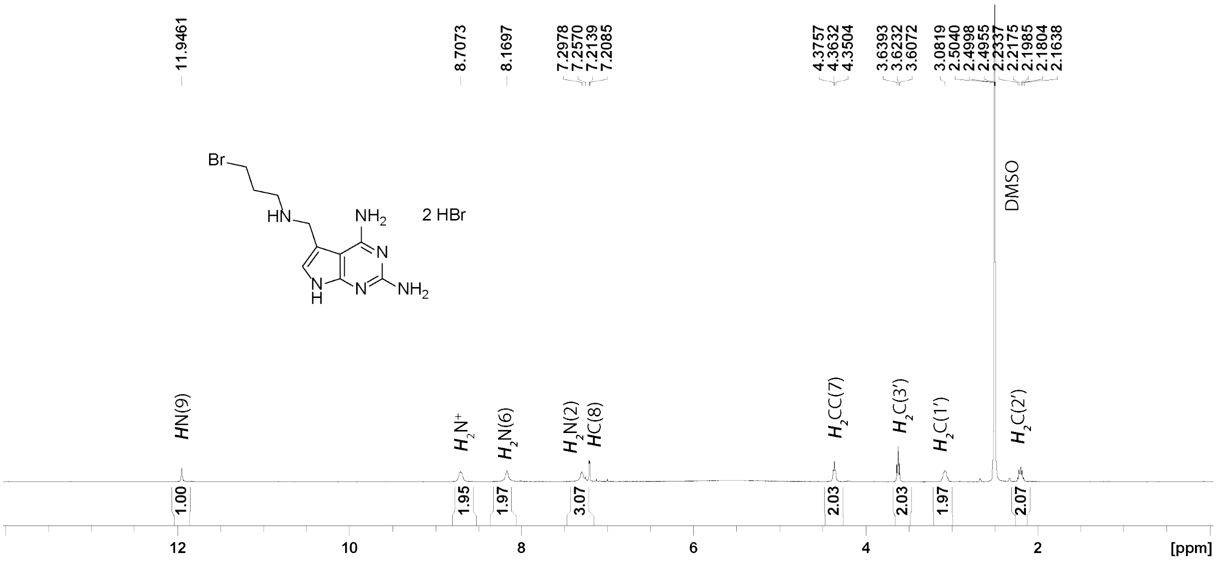

^{1}H-NMR (400 MHz, DMSO-*d6,* 25 °C) spectrum of compound **4c**.

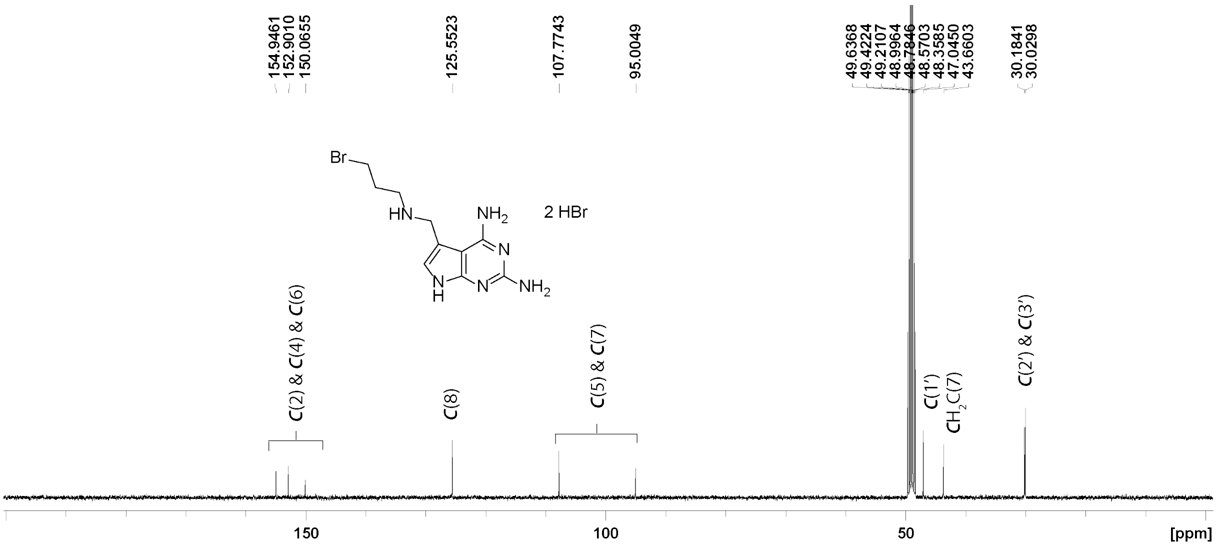

^{13}C-NMR (100 MHz, methanol-*d4*, 25 °C) spectrum of compound **4c.**

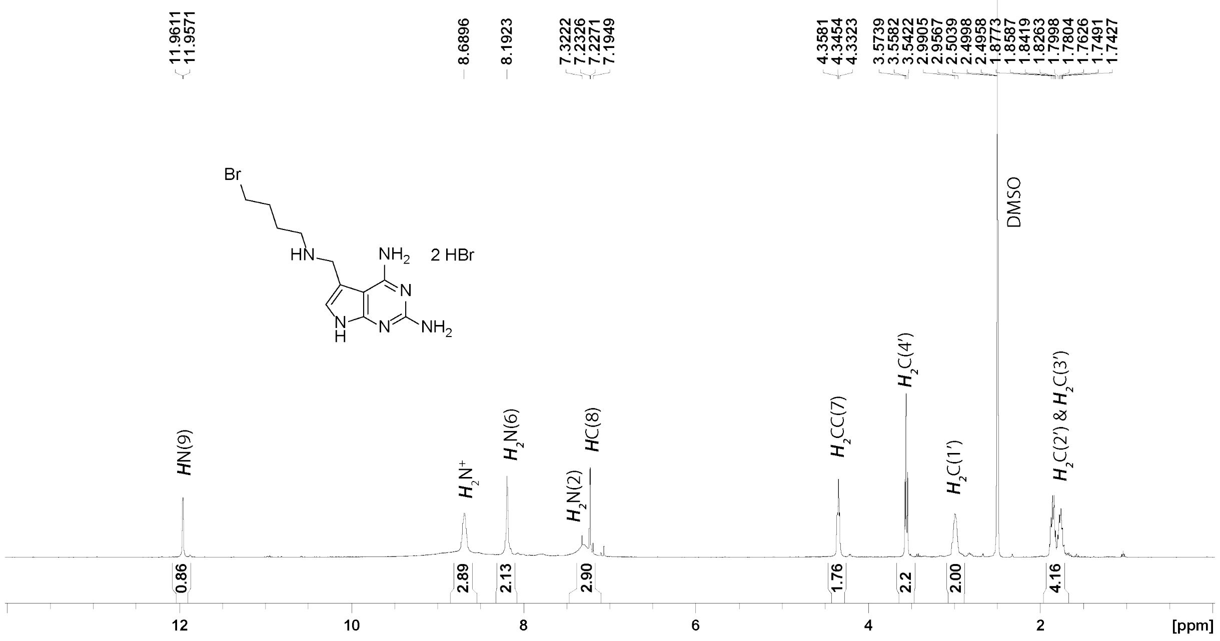

^{1}H-NMR (400 MHz, DMSO-*d*[6], 25 °C) spectrum of compound **4d**.

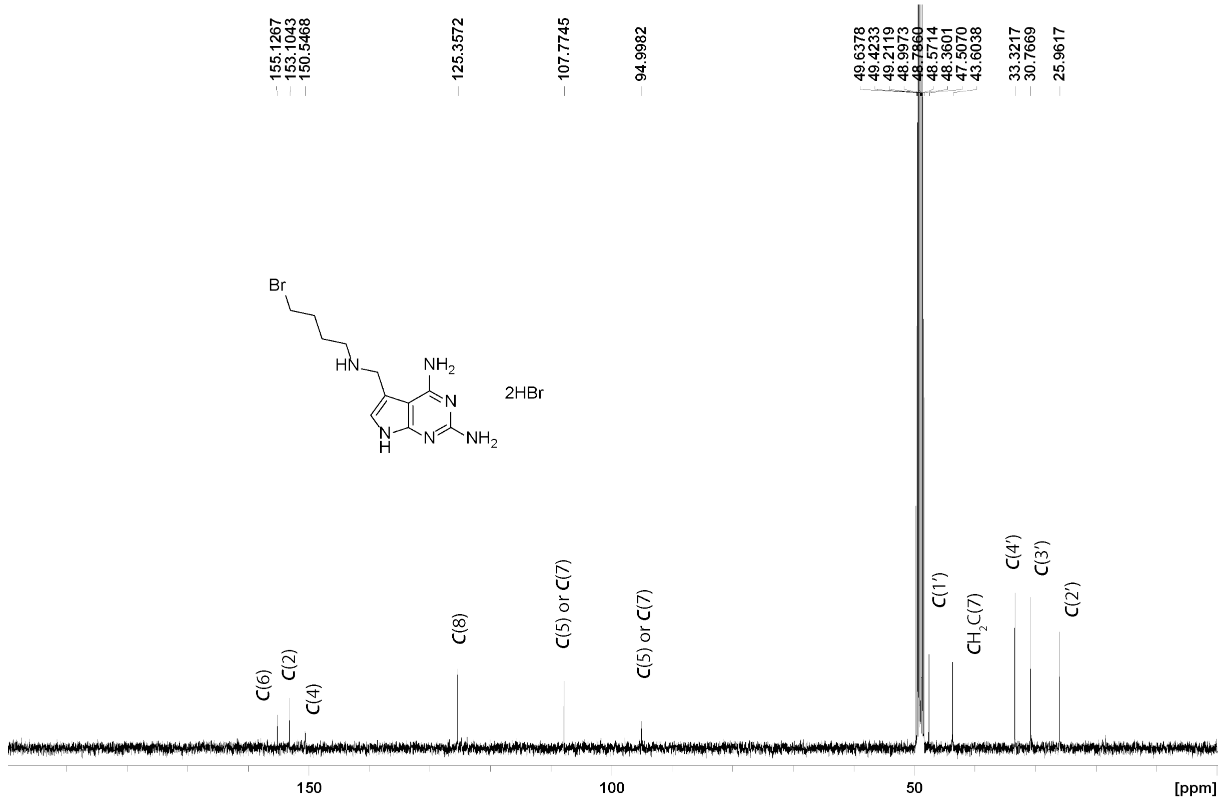

^{13}C-NMR (100 MHz, methanol-*d*[4], 25 °C) spectrum of compound **4d.**

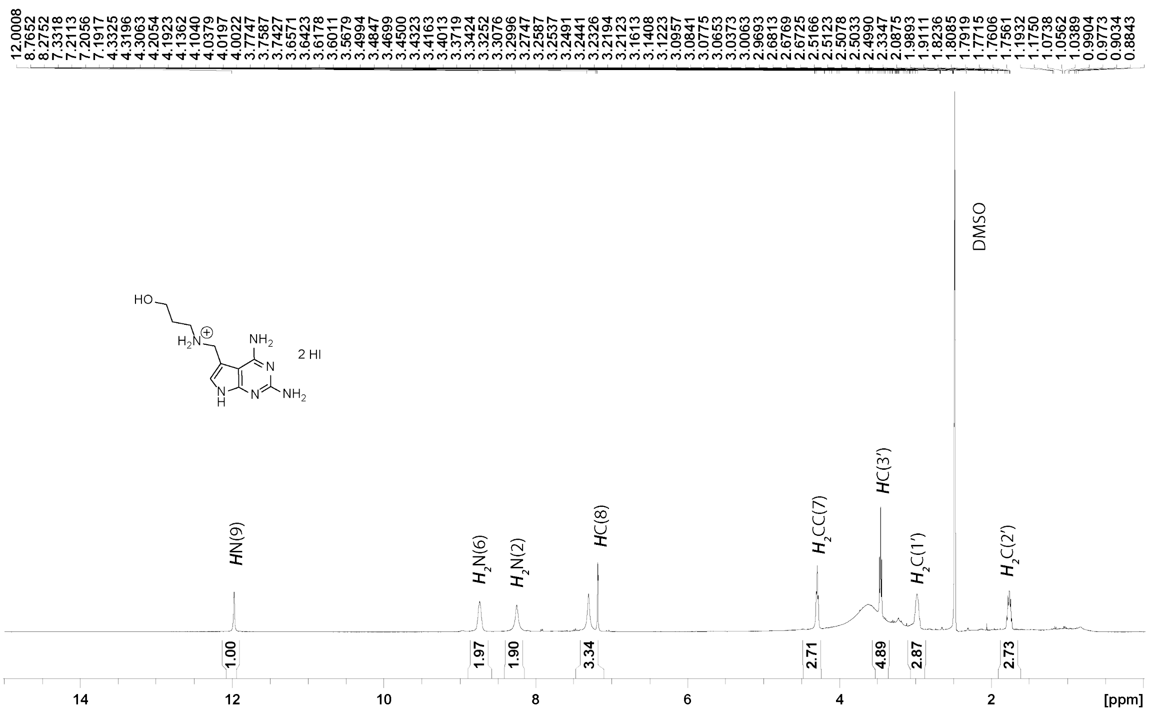

^{1}H-NMR (400 MHz, DMSO-d_6, 25 °C) spectrum of compound **11**.

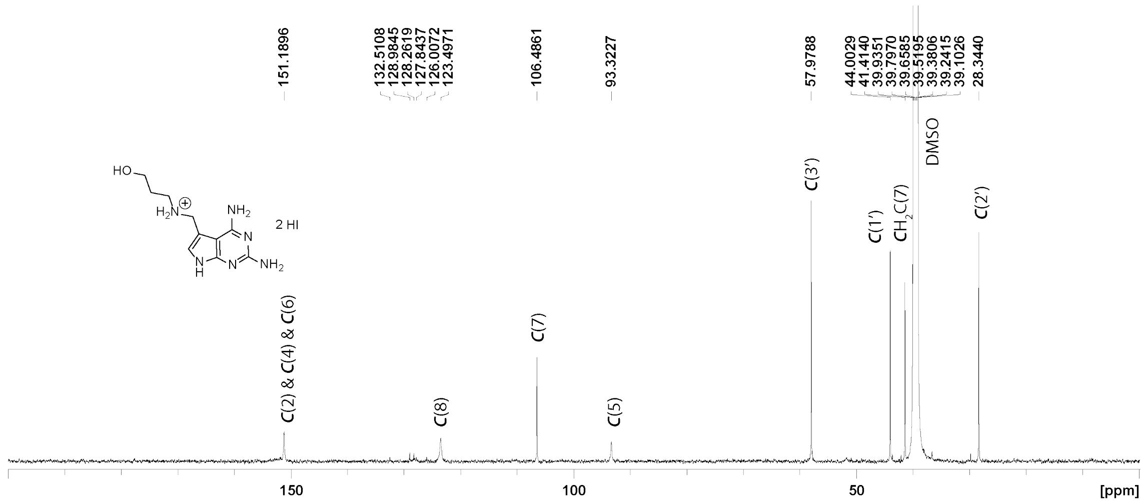

^{13}C-NMR (150 MHz, DMSO-d_6, 25 °C) spectrum of compound **11.**

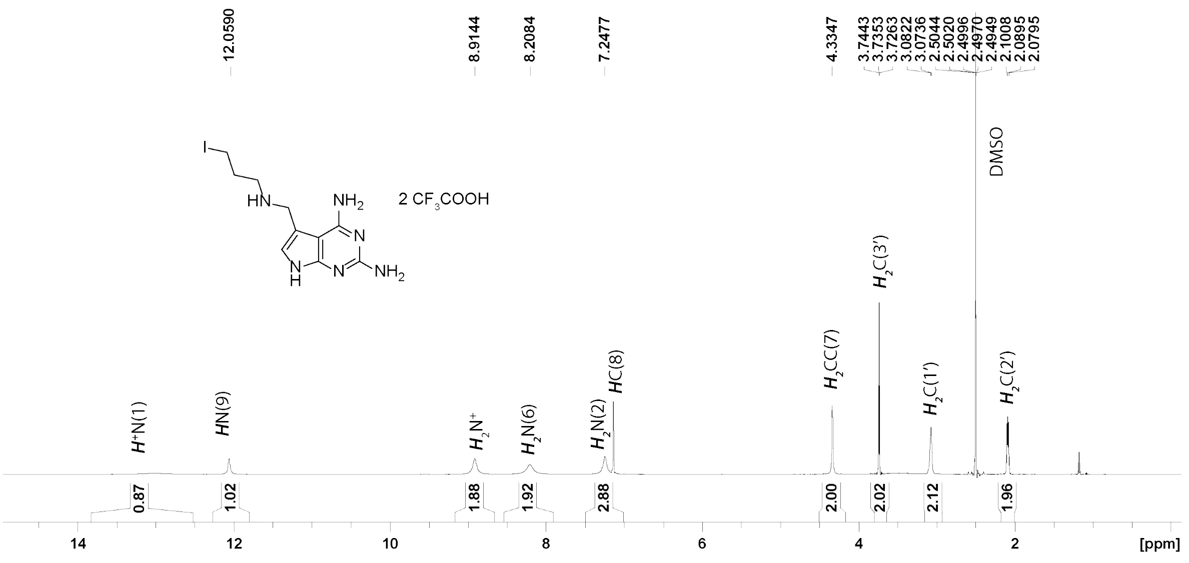

^{1}H-NMR (700 MHz, DMSO-*d*6, 25 °C) spectrum of compound **4e**.

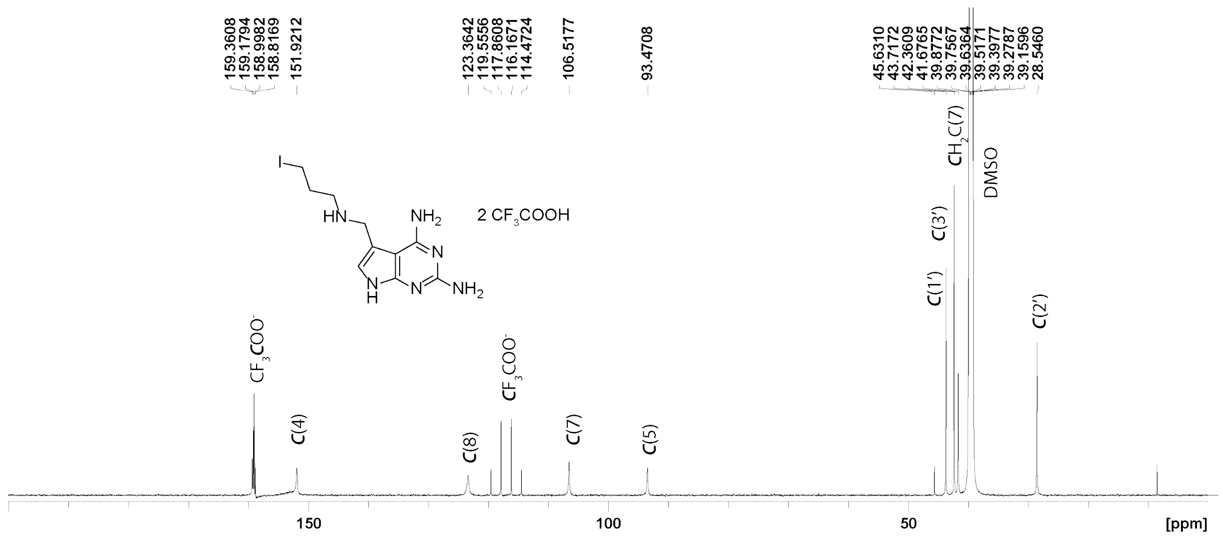

^{13}C-NMR (176 MHz, DMSO-*d*6, 25 °C) spectrum of compound **4e.**

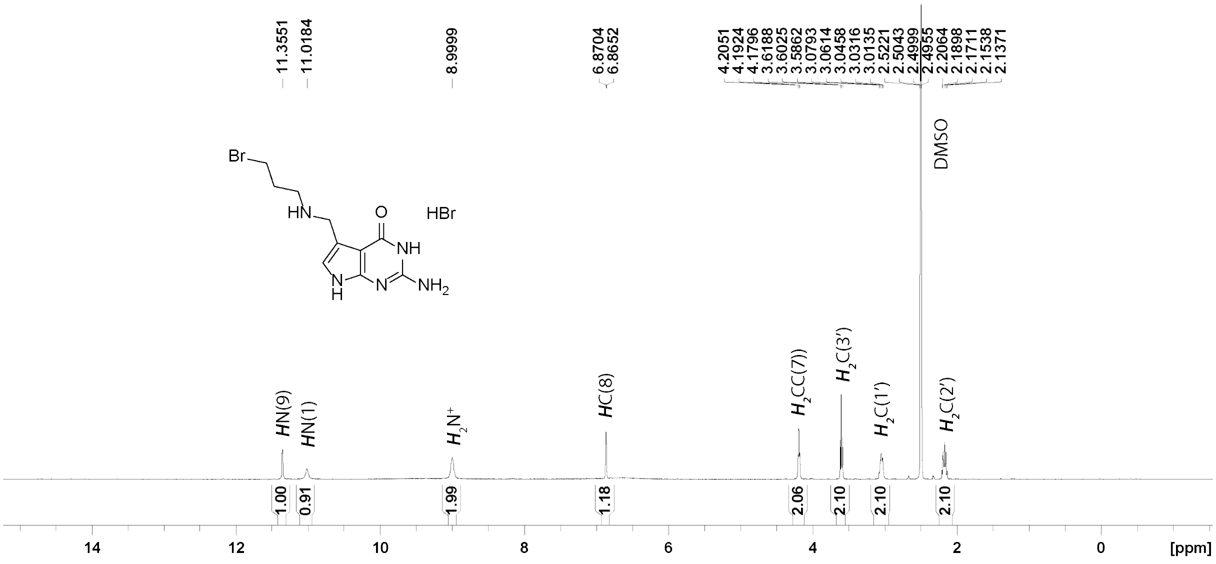

^{1}H-NMR (400 MHz, DMSO-*d6,* 25 °C) spectrum of compound **3a.**

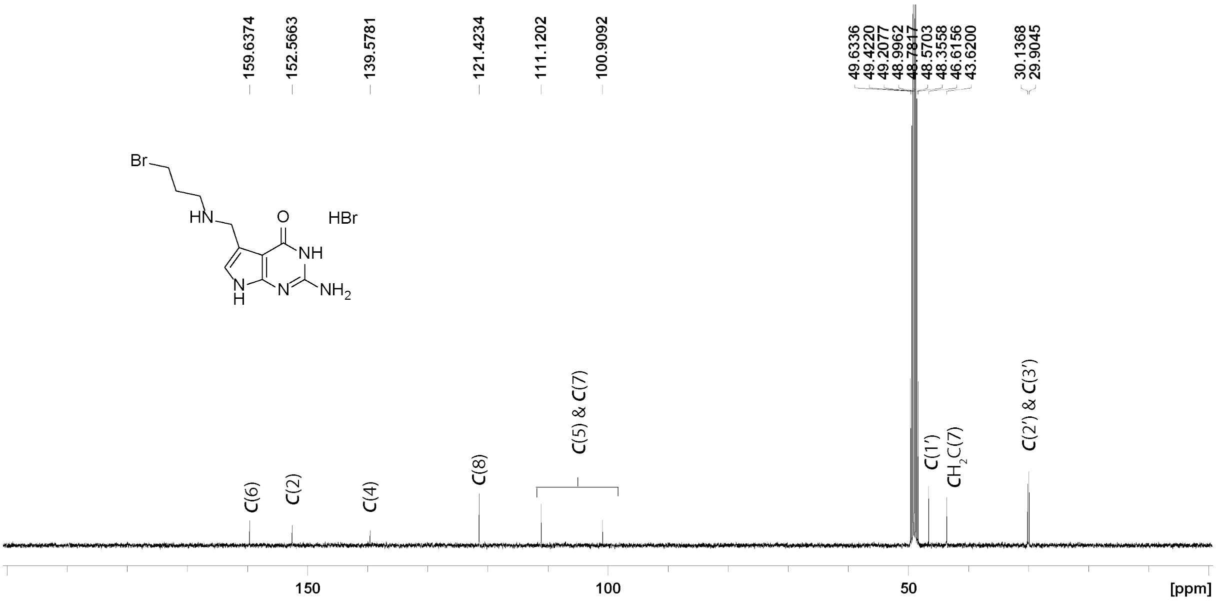

^{13}C-NMR (100 MHz, methanol-*d6,* 25 °C) spectrum of compound **3a.**

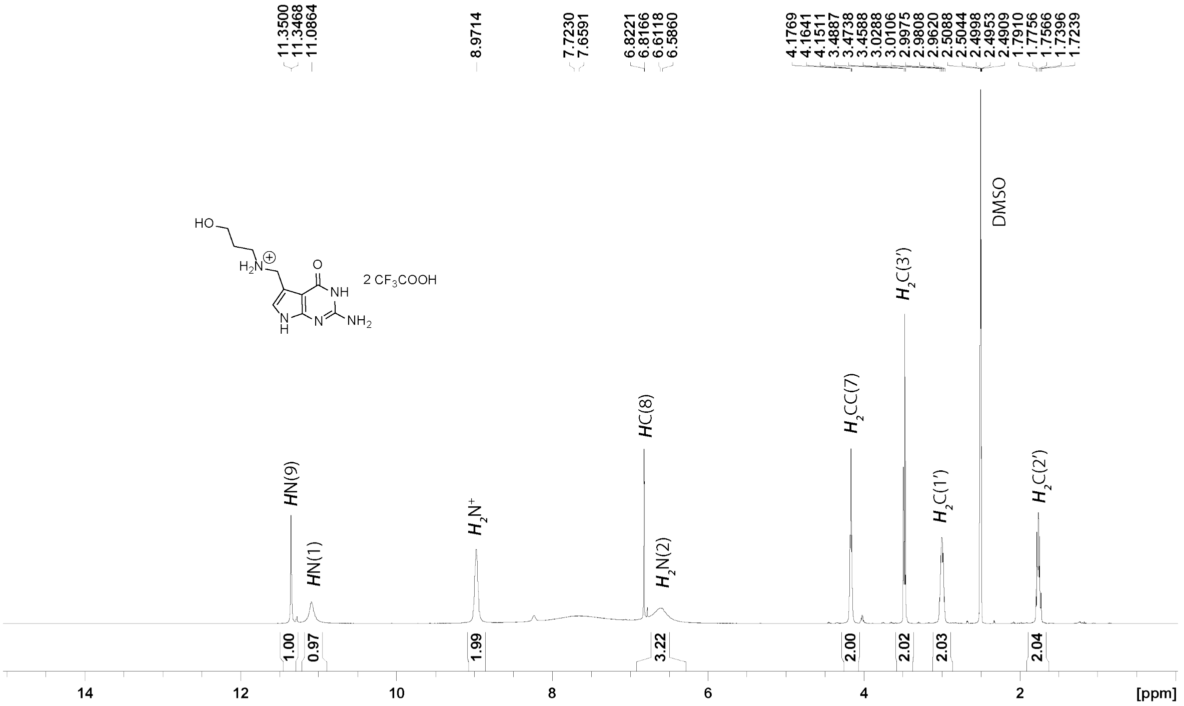

^{1}H-NMR (400 MHz, DMSO-*d6,* 25 °C) spectrum of compound **12.**

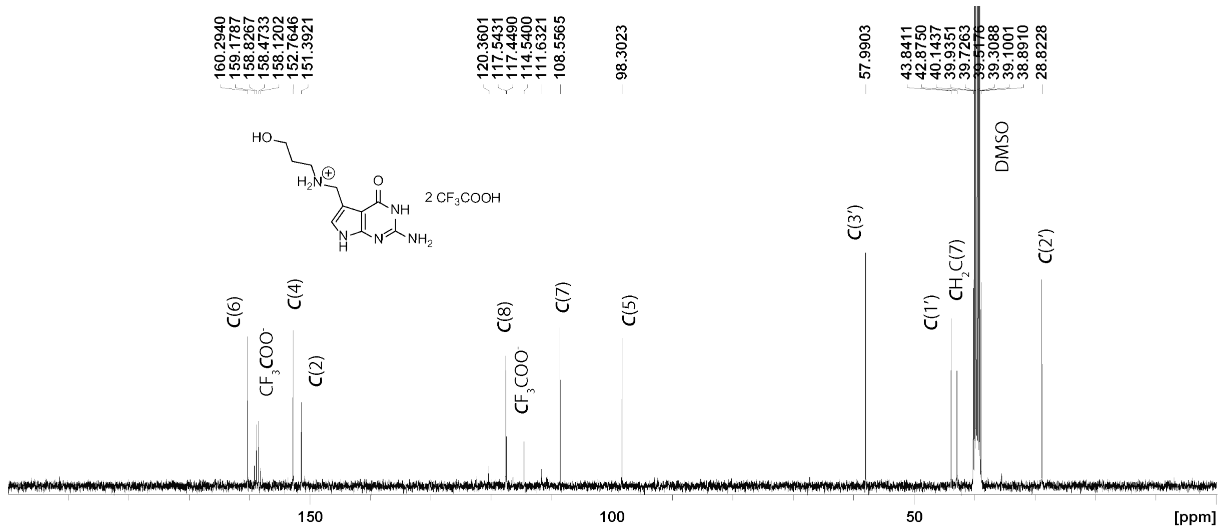

^{13}C-NMR (100 MHz, DMSO-*d6,* 25 °C) spectrum of compound **12.**

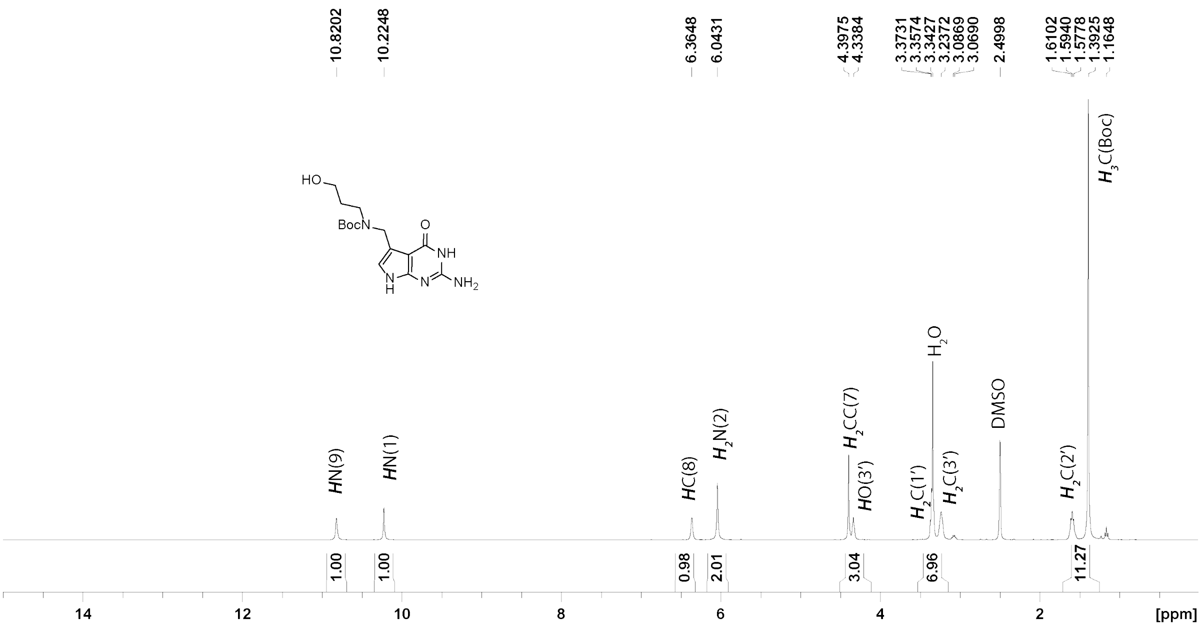

^{1}H-NMR (400 MHz, DMSO-*d6,* 25 °C) spectrum of compound **13.**

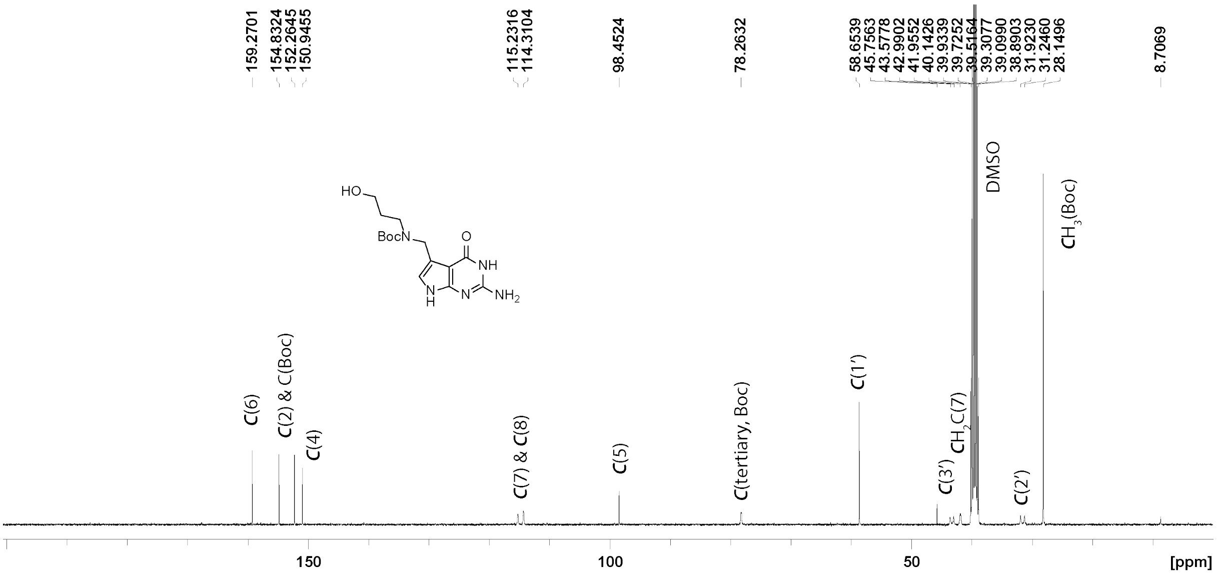

^{13}C-NMR (100 MHz, DMSO-*d6,* 25 °C) spectrum of compound **13.**

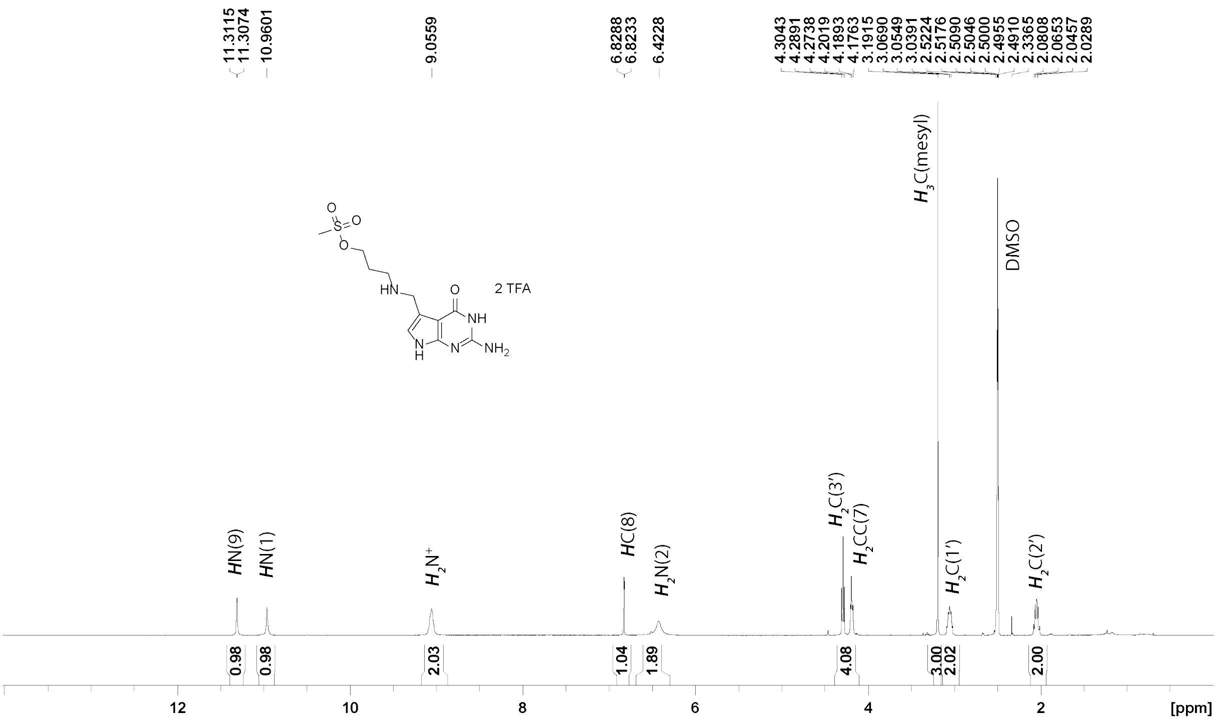

^{1}H-NMR (400 MHz, DMSO-*d6,* 25 °C) spectrum of compound **4b.**

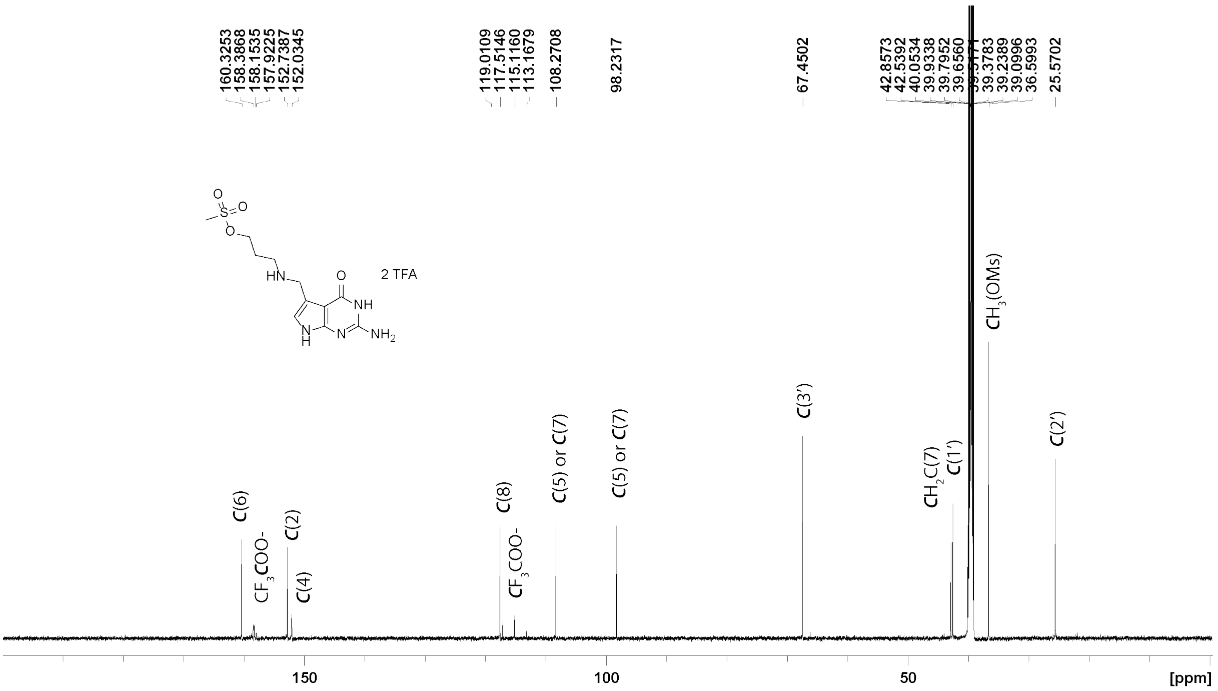

^{13}C-NMR (150 MHz, DMSO-*d6*, 25 °C) spectrum of compound **4b.**

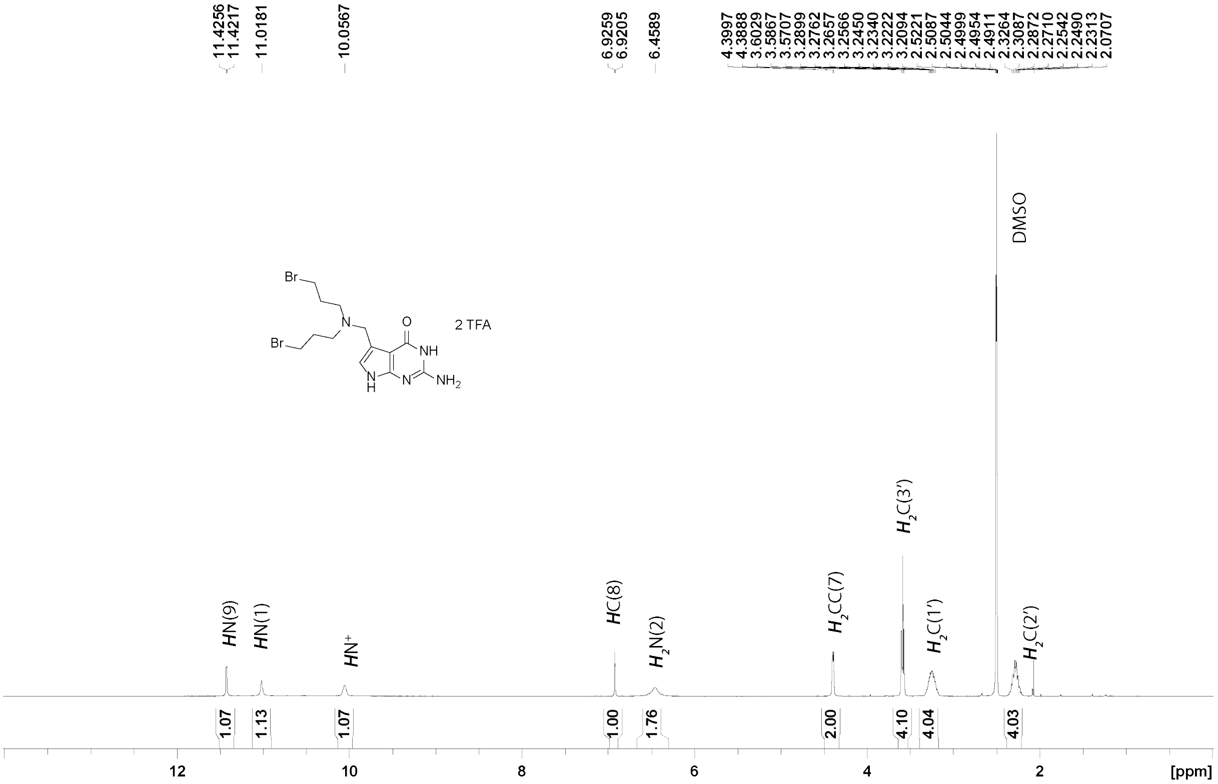

^{1}H-NMR (100 MHz, DMSO-d_6, 25 °C) spectrum of compound **4c.**

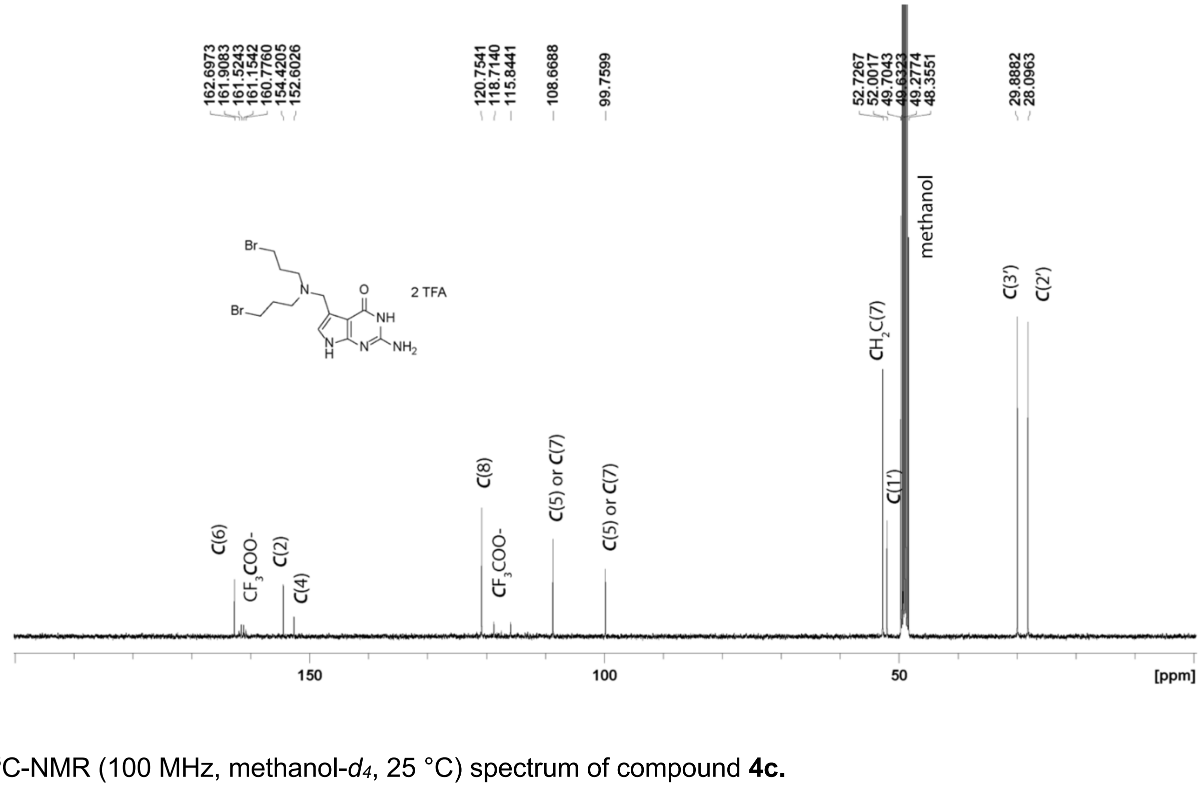

^{13}C-NMR (100 MHz, methanol-d_4, 25 °C) spectrum of compound **4c.**

5. ^{1}H-NMR spectrum of compounds 16

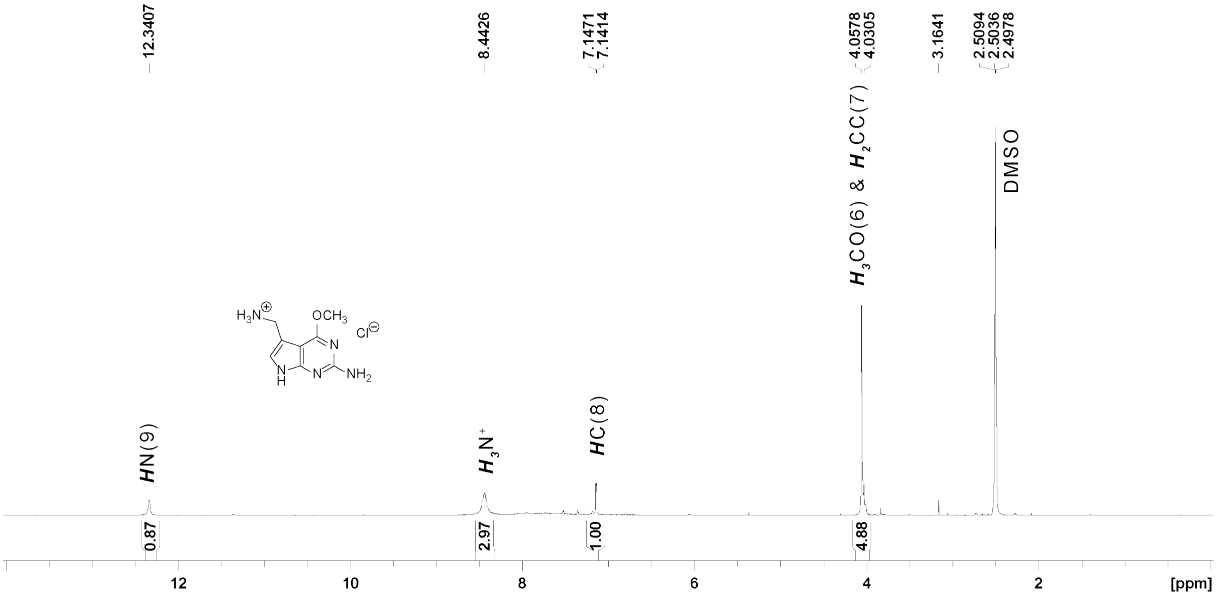

^{1}H-NMR (400 MHz, DMSO-*d6*, 25 °C) spectrum of compound **16.**

6. References

[1] Flemmich, L.; Moreno, S.; Micura, R. *Beilstein J. Org. Chem.* **2021**, *17*, 2295–2301. Doi:10.3762/bjoc.17.147
